# A high-dexterity soft neuroprosthetic hand for daily activities

**DOI:** 10.1038/s41467-026-75105-6

**Published:** 2026-07-04

**Authors:** Ningbin Zhang, Xinyu Yang, Zheng Zong, Yang Yu, Yi Zhao, Rong Bian, Chengru Jiang, Fengjie Shen, Xiangyang Zhu, Guoying Gu

**Affiliations:** 1 State Key Laboratory of Mechanical System and Vibration, School of Mechanical Engineering, Shanghai Jiao Tong University, Shanghai, China; 2 Shanghai Key Laboratory of Intelligent Robotics, Shanghai Jiao Tong University, Shanghai, China; 3 Meta Robotics Institute, Shanghai Jiao Tong University, Shanghai, China

**Keywords:** Mechanical engineering, Biomedical engineering

## Abstract

Robotic prostheses are the primary replacement for upper-limb amputees’ lost hands. Although various neuroprosthetic hands have been developed, featuring both rigid and soft mechanisms, they typically offer grasping-oriented functionality with fewer degrees of freedom (DOFs, ≤6). Herein, we report a dexterous soft neuroprosthetic hand with 11 active DOFs that restores common grasping and fine manipulation, validated by four amputee subjects (aged 38–70 years, male and female). The neuroprosthetic hand integrates soft fingers with thumb-palm articulations for dexterity and adaptivity via a 2-channel myoelectric interface with amplitude-speed decoding, enabling amputees to outperform current prostheses in standardized tests and maintain control robustness. Furthermore, the neuroprosthetic hand improves amputees’ participation in daily activities and interactions, such as hair braiding, pill taking, scissor manipulation, car steering, and continuous bulb screwing, while reducing compensatory body movements. These results demonstrate that the high-dexterity soft-robotic hand enhances both prosthesis versatility and compliant interaction.

## Abstract (web summary)

Robotic prostheses are the main replacement for upper-limb amputees. Existing neuroprosthetic hands, rigid or soft, typically offer grasping-oriented function with ≤6 degrees of freedom (DOFs). Here, the authors report a soft neuroprosthetic hand with 11 active DOFs that restores both grasping and fine manipulation, validated by four amputees (aged 38–70). Using a 2-channel myoelectric interface, it outperforms current prostheses in standardized tests and enables daily tasks like hair braiding, pill taking, scissor use, car steering, and bulb screwing while reducing compensatory movements. This high-dexterity soft hand enhances both versatility and compliant interaction.

## Introduction

Upper-limb amputations, affecting over five million people worldwide due to trauma, disease, and congenital causes, represent a significant global health challenge^1–5^. These individuals urgently require prosthetic hands with near-human dexterity to perform daily activities. This dexterity spans from stable multi-finger grasping to fine dynamic manipulation to natural interaction with humans and environments, which defines the core requirements for general-purpose use^6–8^.

The human hand’s remarkable dexterity, enabled by over 20 DOFs^9^ and inherent compliance, relies on its musculoskeletal and nervous systems to orchestrate key movements like finger flexion, lateral motion, and thumb opposition. Despite ongoing efforts, replicating this dexterity in prosthetic hands remains a fundamental challenge. Most prosthetic hands employ low-DOF mechanisms based on separated rigid components (e.g., motors, linkages, and joints)^2,10–14^, even when strengthened by partially compliant mechanisms^15–19^. In these designs, adding more active DOFs inevitably increases mechanical and control complexity, consequently limiting most prosthetic hands to 6 or fewer DOFs^10^, thereby restricting functionality to basic grasping with few types. Correspondingly, research efforts in advanced neural control strategies^20–22^, neuromotor synergy^23^, interface implementation^24–28^, and sensory feedback^29–32^ for prosthetic hands continue to focus predominantly on grasping. While a recently developed 19-DOF robotic hand has demonstrated capabilities beyond simple grasping, it is designed with an inherently rigid mechanism, and it is challenging to integrate a neural control interface for hand amputation applications^33^.

Soft robotics^34^, an alternative paradigm for designing robotic hands^35–42^ using soft material-based structures, has recently emerged as a promising approach to overcome the limitations of “rigid” solutions. Our previous study^43^ has presented a lightweight soft neuroprosthetic hand that employs continuously deforming compliant structures to achieve passive shape conformance without requiring complex control. Similar to other reported studies^40,44,45^, the robotic hand mechanism maintains no more than 6 DOFs. Although some preliminary concepts of high-DOF ( > 6) soft-rigid hands have emerged^46^, to date, designing and implementing an effective high-DOF hand prosthesis for amputees with human-like versatility beyond basic grasping remains an unresolved challenge.

**Fig. 1:**
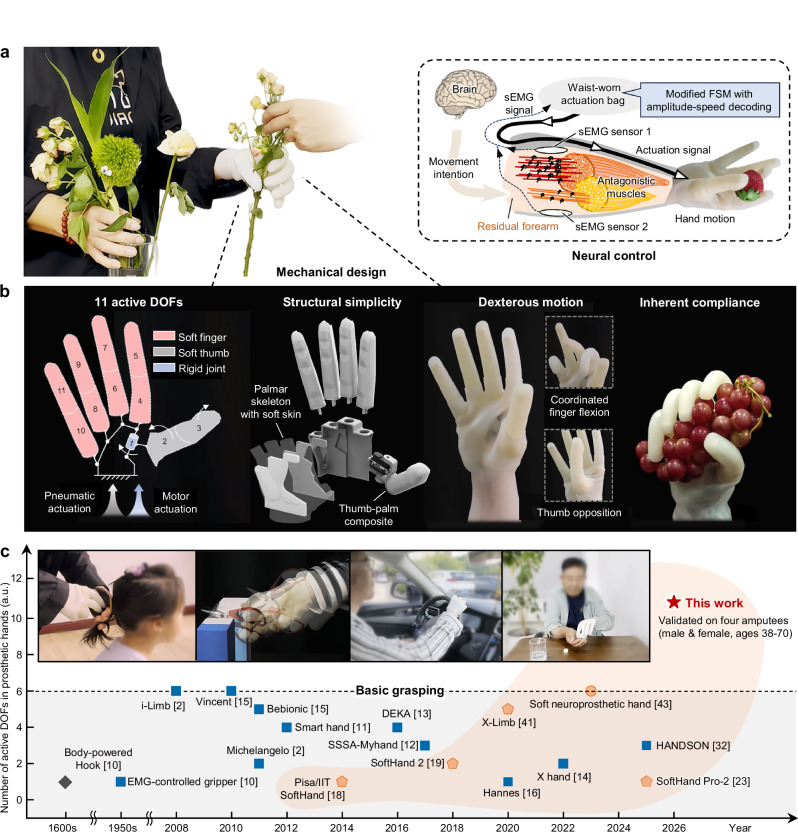
High-dexterity soft neuroprosthetic hand design and capabilities. **a** Overview of the soft neuroprosthetic hand. The neuroprosthetic hand integrates an anthropomorphic soft-rigid hand mechanism, enabling the amputee to intuitively control it using a two-channel sEMG neural interface with a modified finite-state machine (FSM) decoding algorithm. The sEMG signals are captured from two antagonistic muscle groups on the residual limb, with actuation components and electronics contained within a waist-worn bag. **b** Mechanical design and capability features. The hand mechanism has eleven active degrees of freedom (DOFs) and inherent compliance. Actuated by pneumatic pressure and a micromotor, the eleven DOFs are distributed across a thumb-palm composite and four fingers, generating thumb opposition and coordinated finger flexion. **c** Comparisons of our prosthetic hand with existing designs. With its eleven active DOFs and inherent compliance, the prosthetic hand supports a wide range of daily activities for amputees, including grasping, manipulation, and adaptive interaction, addressing the long-standing challenge in prosthetic hand design dating back to the early centuries. Compared to current solutions that are either functionally limited to basic grasps or rendered impractical by their complexity (detailed in Supplementary Tables 1–3), the neuroprosthetic hand demonstrates real‑world functionalities previously unattainable, thereby paving the way for more versatile and natural prosthetic applications worldwide. Blue square, rigid linkage-driven designs. Orange pentagon, tendon-driven compliant designs. Orange circle, pneumatic-driven soft designs.

Here, we introduce a high-dexterity soft neuroprosthetic hand that restores commonly-used grasping and manipulation functionalities for amputees with upper-limb amputation in daily life (Fig. 1a and Supplementary Video 1). Its dexterity arises from a coherent soft-rigid hybrid design of pneumatic soft fingers with motor-driven mechanisms, delivering 11 active DOFs and inherent compliance (Fig. 1b). To translate the mechanical dexterity into functional commands, we develop a robust decoding algorithm that is seamlessly deployed through a minimalist 2-channel surface electromyography (sEMG) interface, enabling intuitive neural control by amputee users (Fig. 1a). The hand’s effectiveness is experimentally validated through numerous standard tests and real-world tasks from daily activities, along with positive subjective user evaluations, demonstrating its capabilities in versatile adaptive grasping, natural dynamic manipulation, and intimate social interaction (Fig. 1c). Compared to current solutions and our previous work^43^ for basic grasping, the neuroprosthetic hand achieves functionalities that were previously unattainable, pointing toward a future for general-purpose neuroprosthetic hands (Supplementary Tables 1–3).

## Results

### Design and fabrication

**Fig. 2:**
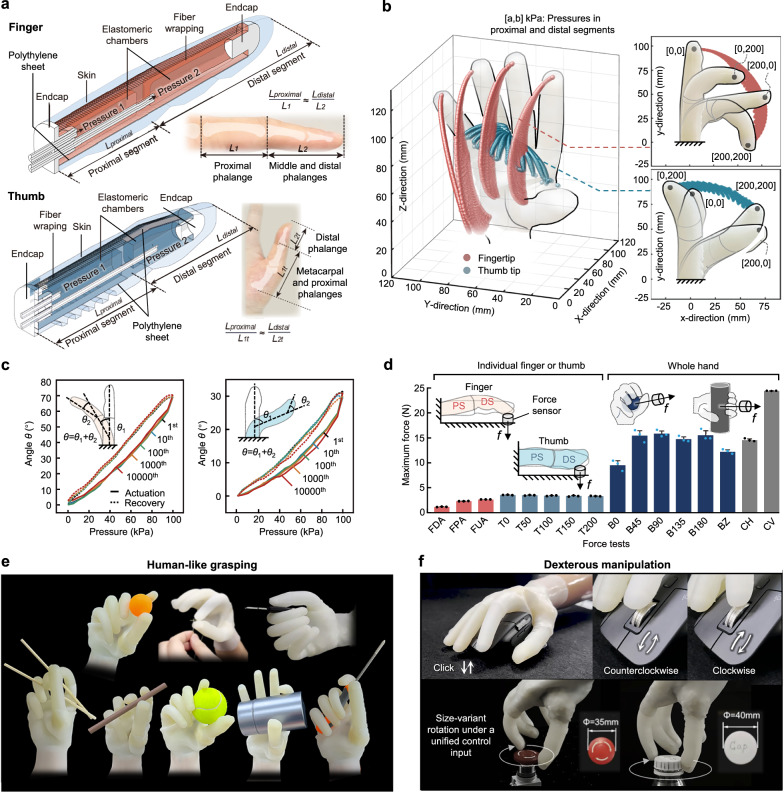
Mechanical structure and hand performance. **a** Structure of the soft finger and thumb. Each digit (finger or thumb) is structured as a fiber-reinforced soft actuator containing two independent chambers to achieve desired workspace, with segment length ratios designed to mimic the human anatomy. For the finger, a single polyethylene sheet spans the palmar side of both segments to act as a strain-limiting layer. For the thumb, two separate strain-limiting layers are used, with one on the palmar side and one on the dorsal side. **b** Active workspace of the hand mechanism, which is generated by all the fingertip trajectories under actuation. Each finger segment is actuated with pneumatic pressure varying from 0 kPa to 200 kPa. The rigid joint of the thumb-palm composite is actuated from 0° to 60°. **c** Durability of the finger (left panel) and the thumb (right panel) over 10,000 actuation cycles. **d** Force capabilities of the hand mechanism. Abbreviations (FDA, FPA, FUA): fingertip forces measured in distal-only, proximal-only, and unified actuation modes. Abbreviations (T0, T50, T100, T150, T200): thumb’s fingertip force with its distal segment pre-inflated to 0, 50, 100, 150, 200 kPa. Abbreviations (B0, B45, B90, B135, B180): omnidirectional hand forces measured by pulling a power-grasped ball of 60-mm diameter at angles of 0°, 45°, 90°, 135°, 180°. Abbreviation (BZ): the force measured by dragging a ball (60 mm diameter) vertically away from the hand mechanism. Abbreviation (CH, CV): the forces measured by pulling a cylinder (55 mm diameter) away from the hand mechanism in horizontal and vertical directions. Each test consists of *n* = 3 measurements (error bars: s.d.). **e** Representative grasp types toward replicating the human hand. **f** Demonstrations of coordinated, dynamic manipulation capabilities.

The neuroprosthetic hand’s mechanical structure comprises four soft fingers and a thumb-palm composite mounted on a palmar skeleton covered with elastic skins (Fig. 1a). Each digit (finger or thumb) features a multi-layer elastomeric structure with two independently actuated serial segments for multi-articulated kinematics and tissue-like compliance (Fig. 2a). With individual pneumatic actuation, the two finger segments generate programmed codirectional bending, replicating the independent yet complementary kinematics of the metacarpophalangeal (MCP) and proximal interphalangeal (PIP) joints in the human hand^9^. The dimensions of finger segments are determined through parametric studies (Supplementary Fig. 1, Methods, and Supplementary Text 1). Given the trade-off between functional benefits and structural simplicity, lateral movements of fingers are achieved through passive compliance rather than active control (Supplementary Fig. 2). The thumb-palm composite employs a 3-DOF soft-rigid hybrid mechanism, combining a distal soft thumb with two pneumatic segments for reverse-bending flexibility and a proximal motor-driven joint for stable circumduction. Thus, the hand mechanism can generate diverse grasps and manipulations through the coordinated motion of the thumb-palm composite and four fingers, with safe and adaptive interaction owed to the embodied flexibility.

Our neuroprosthetic hand is fabricated using a scalable process that combines soft casting^34^ (for the fingers, thumb, and skins), stereolithography and selective laser sintering 3D printing (for the palmar skeleton, rigid components, and casting molds), and simple assembly. Detailed fabrication processes are provided in Supplementary Fig. 3 and “Methods”. Owing to the modular and mechanically simple design, the hand mechanism itself weighs 242 g, lighter than the average human hand (about 400 g)^9^. The hand mechanism connects to a customized arm socket fitted to the amputee’s residual limb. The socket incorporates a passive wrist and two commercial sEMG sensors (Danyang Inc., each 10 g) that contact the skin to non‑invasively acquire the amputee’s motion intentions from the flexor and extensor muscles, respectively (Supplementary Fig. 4). These signals are further decoded into the neuroprosthetic hand’s control commands through a modified finite state machine (FSM)^47^ to execute various grasps and manipulations. These commands, along with pneumatic pressure, are delivered via electrical wires and tubes from a waist-worn bag, which contains actuation and power components (e.g., micro pumps, valves, processors, electronics, and batteries, detailed in Supplementary Table 4). The total system weight, including the hand mechanism and waist-worn bag, is 703 g (Supplementary Table 1). To promote wearability, the electrical wires and tubes are enclosed in a compact sleeve, which is secured to the amputee’s clothing via adjustable flexible anchors (Supplementary Fig. 5). The overall operational noise is at a low level (≤ 48 dB at 0.25 m, Supplementary Fig. 6).

### Mechanical performance

We first evaluate the kinematic performance of the hand mechanism. An active workspace is defined to quantify the fingertip reachability under actuation (0–200 kPa per finger segment; 0°–60° rotation of motor-actuated joint), with fingertip trajectories recorded by visual tracking (Fig. 2b). Experimental details are provided in Supplementary Fig. 7 and “Methods”. Within the active workspace, each fingertip sweeps out a crescent-like area through codirectional bending, and the thumb tip generates a rhombic-like area via contradirectional bending. Actuation of the palm joint further expands the thumb’s workspace, producing overlap with those of fingers to enable fundamental thumb opposition (Supplementary Fig. 8). This programmability in active workspace allows for delicate pose control, whereas the hand’s intrinsic compliance broadens its functional range. Moreover, we conduct durability tests exceeding 10,000 actuation cycles, examining both free motion of the finger/thumb (Fig. 2c) and coordinated grasping with object contact (Supplementary Fig. 9). The results demonstrate good, consistent performance with negligible degradation (< 5% motion error).

We then comprehensively characterize the force performance (Fig. 2d) and functional capabilities of the hand mechanism (see details in Supplementary Figs. 10, 11). The maximum tip forces are 2.6 ± 0.01 N for the finger and 3.5 ± 0.05 N for the thumb to enable safe and adaptive interaction (mean ± s.d., *n* = 3 tests). Measured stiffnesses (61.0 ± 2.2 N/m laterally for the fingers; 163.5 ± 10.9 N/m on average for the thumb-palm composite across four directions; mean ± s.d., *n* = 3 tests) confirm enhanced stability compared to the prior design^43^ (24.6 ± 3.8 N/m; 14.9 ± 1.9 N/m). Experimental data are provided in Supplementary Table 5. Measured maximum pull-out forces (15.8 N for omnidirectional sphere grasp; 24.4 N for cylinder grasp, remaining at 23.7 N after 10,000 cycles) exceed the ~10 N requirement for human hands during commonly-used daily tasks^7^. The hand mechanism can replicate 33 grasp types in the human GRASP Taxonomy^7^ via actuation programming and inherent self-adaption (Fig. 2e, Supplementary Fig. 12 and Supplementary Table 6). The finger can apply tangential forces (pushing or dragging) and normal forces (pressing). This capability is exemplified by the hand mechanism accomplishing a multimodal manipulation task involving bidirectional scrolling and clicking of a computer mouse. Furthermore, by combining finger‑thumb coordination with inherent omni-directional compliance, our neuroprosthetic hand generates adaptive rotational torques to provide functional scalability without reprogramming, as shown in the manipulation of objects with varied diameters (Supplementary Video 2 and Fig. 2f).

### Neural control interface

**Fig. 3:**
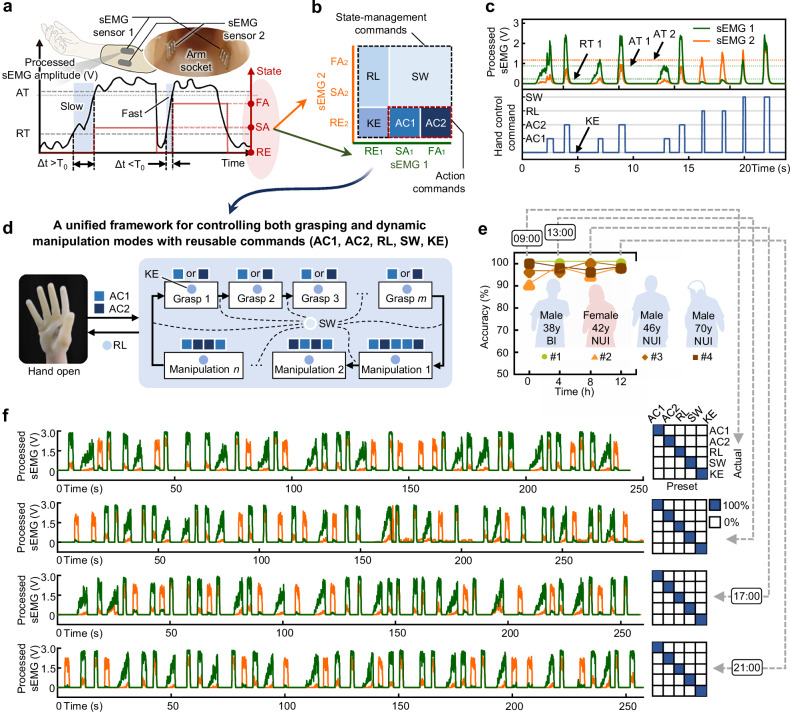
Neural control scheme and resulting performance. **a** Measurement and decoding of the two-channel sEMG signals from the subject’s residual limb. Taking channel sEMG 1 as an example, the signal is processed and decoded based on its amplitude and speed of amplitude variations. Action threshold (AT) and rest threshold (RT) are the preset thresholds of the signal amplitude. Fast active (FA), slow active (SA), and rest (RE) are the corresponding three states. Δ*t*, the time taken for the amplitude to rise from RT to AT. T_0_, predefined time threshold. **b** Mapping matrix that converts the two-channel signal states into hand control commands: action 1 (AC1), action 2 (AC2), release (RL), switch (SW), and keep (KE). **c** Continuous sEMG signals recorded from an amputee subject and the corresponding hand control commands. **d** A unified framework for the user controlling the neuroprosthetic hand to perform both grasping and manipulation modes via a modified FSM. **e** Average control accuracy during 12-hour use of four amputee subjects, tested every 3-hour with *n* = 50 randomly preset commands per test. UNI, unilateral amputation. BI, bilateral amputation. Letter ‘y’, years old. Sign ‘#’, amputee identification number. **f** Time-varying sEMG signals (left) and the resulting confusion matrix (right) of subject #1 during the tests. The preset control commands are detailed in Supplementary Table 8. Subject-specific sEMG signal thresholds are provided in Supplementary Table 9.

Our sEMG decoding algorithm builds upon the FSM strategy, a robust and widely adopted approach in neuroprosthetic hand control^47^. However, most current implementations rely on a single amplitude threshold, which restricts control to binary on/off grasping (Supplementary Text 2), thus fundamentally incompatible with the high mechanical dexterity. In our modified FSM, three distinct thresholds are preset for the sEMG signal from each sensor (Fig. 3a): two amplitude thresholds (AT and RT, for action and rest respectively; AT > RT) and one time threshold (T_0_). These thresholds are all tailored to each amputee subject, as detailed in Supplementary Fig. 13. Using them, three signal states are defined: ‘Rest (RE)’ when the amplitude remains below RT, ‘Slow Active (SA)’ when it rises from RT to AT over period longer than T_0_, and ‘Fast Active (FA)’ when it completes this rise within a time less than T_0_ (Supplementary Fig. 14). Thus, the signal states of the two sEMG sensors (i.e., RE_*i*_, SA_*i*_, FA_*i*_, where *i* = 1 denotes flexor muscles, and *i* = 2 extensor muscles) form a state matrix and theoretically yield nine distinct control commands. In practice, factors like signal crosstalk^48^ render some commands physiologically indistinguishable. To address this, we consolidate them to yield a reduced hand-control matrix for practical use (Fig. 3b). Amputee subjects typically master these commands within 5–10 minutes of training (“Methods”) and achieve reliable control (Fig. 3c and Supplementary Video 3).

This modified FSM algorithm allows the hand mechanism to cycle through various specific motion modes within a unified control framework (Fig. 3d). These modes functionally fall into the two broad categories: grasping and manipulation. Within any specific motion mode, the states [SA_1_, RE_2_] and [FA_1_, RE_2_] respectively map to the commands: primitive action 1 (AC1) and primitive action 2 (AC2). A primitive action is defined here as an elementary and reusable unit that constitutes the motion mode itself or can be combined to form more complex behaviors. The state [RE_1_, SA_2_ or FA_2_] maps to the command Release (RL), returning the hand to its home position. The state [RE_1_, RE_2_] maps to the command Keep (KE), which indicates no new movement. The state [SA_1_ or FA_1_, SA_2_ or FA_2_] maps to the command Switch (SW), thereby initiating a transition to the next motion mode. In a grasping motion mode, only a single primitive action is employed. Therefore, AC1 and AC2 are functionally equivalent and can be merged. In a manipulation mode, AC1 and AC2 serve as functional components that can be flexibly called upon to accomplish the task. RL, KE, and SW are common to both grasping and manipulation modes. Supplementary Video 4 and Supplementary Fig. 15 show an amputee intuitively controlling the prosthetic hand to perform desired grasping and manipulation modes. The motions of the fingers and thumb are driven by a pneumatic timing control system, which governs the activation of pumps and valves. If needed, the system allows the user to progressively regulate pneumatic pressure through repeated commands, which enables more delicate grasp adjustment and the execution of complex manipulation tasks (Supplementary Fig. 16 and Supplementary Video 5).

Furthermore, we evaluate the control effectiveness under user-induced fatigue and skin-electrode impedance fluctuations (Fig. 3e and Supplementary Fig. 17). In these tests, four amputee subjects (including male and female, young and old, aged from 38 to 70, unilateral and bilateral amputation) continuously wore the neuroprosthetic hand for 12 hours. Detailed subject profiles are shown in Supplementary Table 7. Each subject repeatedly performed 50 preset control commands (5 types including AC1, AC2, RL, SW and KE; 10 commands per type) at four discrete time points — 09:00, 13:00, 17:00, and 21:00 — spaced 4 hours apart (Supplementary Table 8). Notably, the subjects did not retrain during this 12-hour period (see Methods and Supplementary Table 9). The results show 100% accuracy for one subject and near-100% performance in the other three subjects (mean ± s.d., 95 ± 3.3%, 97.5 ± 1.7%, 98 ± 1.4%). This consistently reliable performance is maintained under varying skin impedance, as measured periodically over a 12-hour period (Fig. 3f and Supplementary Fig. 18), further demonstrating the practical utility of the neural control interface.

### Standard grasping tests

We evaluate the grasping performance of our neuroprosthetic hand using standardized tests on the four subjects. Prior to the tests, all subjects have provided informed consent after being briefed on the test procedures. Each subject completed a practice session to ensure familiarity and consistency with the testing protocol. The test details are provided in “Methods”.

**Fig. 4:**
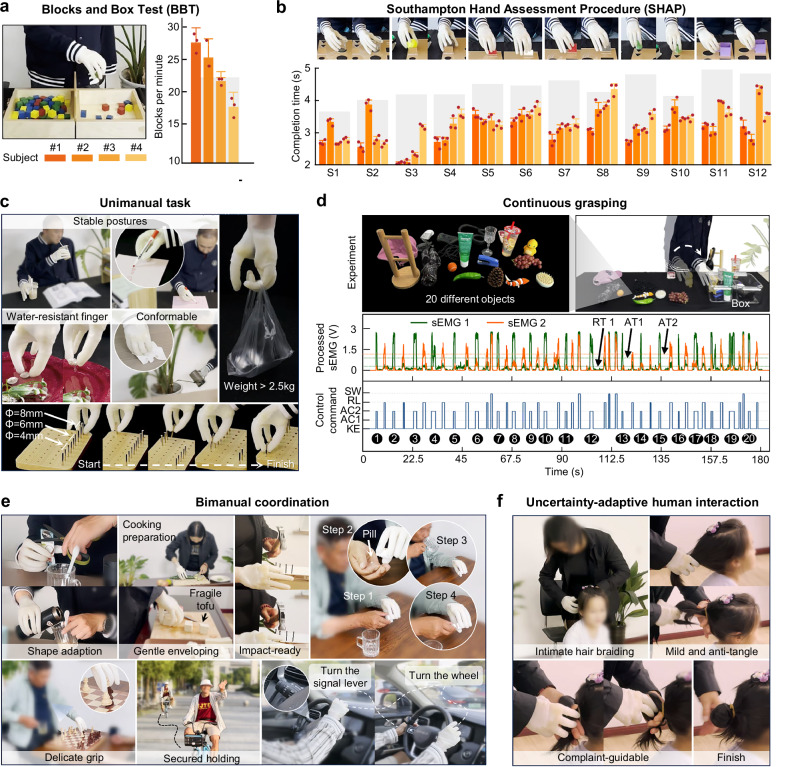
Restoration of versatile grasping capabilities for amputees. **a** Grasping performance evaluation using the Blocks and Box Test (BBT) in 4 subjects. The test for each subject consists of *n* = 3 trials (error bars: s.d.). The test results are compared with the performance benchmark obtained from references^49–51^. **b** Grasping performance evaluation using the Southampton Hand Assessment Procedure (SHAP) in 4 subjects. The test for each subject consists of *n* = 3 trials (error bars: s.d.). The corresponding performance benchmark is obtained from reference^52^. **c** Demonstration of restored unimanual grasping functions. **d** Continuous grasp-and-place of 20 objects into boxes, enabled by sEMG-decoded commands and inherent compliance. The objects vary in shape, dimensions, weights, and textures. Numbered black circles indicate the grasping actions. **e** Demonstration of restored bimanual operation with the neuroprosthetic hand and the healthy hand for overall upper-limb function improvement. **f** Demonstration of a subject seamlessly using the neuroprosthetic hand with the healthy hand to do a hairstyle for her daughter, showcasing fluid bimanual cooperation in a familial, adaptive interaction.

The Box and Blocks Test (BBT)^49^ is employed to evaluate the gross grasp capabilities. In this test (Supplementary Fig. 19a–e and Supplementary Video 6), subjects grasp cubic blocks one by one and transporting them across a partition between boxes within one minute. Figure 4a shows that the BBT scores (i.e., the numbers of blocks transported) of the four subjects are 28.7 ± 1.2, 26.3 ± 1.9, 22.7 ± 0.5, and 18.7 ± 1.2, respectively (mean ± s.d., *n* = 3 tests per subject). The complete test scores are detailed in Supplementary Table 10. These scores meet or exceed the performance benchmark for existing neuroprosthetic hands (14.3 to 24 blocks per minute^49–51^). Compared to our previous work^43^, our neuroprosthetic hand shows a notable increase in BBT scores (Supplementary Table 11). This can be attributed to the stable pinch enabled by the dual-segment finger design (Supplementary Fig. 20), which addresses the fingertip pinch instability^42^.

The Southampton Hand Assessment Procedure (SHAP)^52^ is employed to evaluate the comprehensive grasping function for varied objects (Supplementary Fig. 19f–j, Supplementary Video 7 and Supplementary Table 12). All four subjects perform the following tasks (*n* = 3 trials per subject), and their completion times are recorded: S1-power light, S2-power heavy, S3-spherical light, S4-spherical heavy, S5-tip light, S6-tip heavy, S7-extension light, S8-extension heavy, S9-tripod light, S10-tripod heavy, S11-lateral light, and S12-lateral heavy. A shorter completion time indicates better performance. Figure 4b and Supplementary Table 13 show that the completion times for most tasks across all four subjects are below performance benchmarks^52^. Compared to our previous work^43^, this neuroprosthetic hand shows reduced completion times across various tasks and enables all subjects to complete tasks S10, S11, and S12, which are previously unachievable (Supplementary Table 14).

### Grasping versatility across daily activities

We next demonstrate the grasping versatility in daily activities, ranging from essential life skills to social interactions. The sEMG thresholds remained fixed for each subject throughout all experiments. The properties of the grasped objects are shown in Supplementary Table 15. The neuroprosthetic hand enables a bilateral amputee to perform a variety of unimanual tasks, such as securing a deformable cup without crushing it, turning pages by leveraging the neuroprosthetic skin’s inherent friction, stabilizing a pen to write, holding a watering can, conforming to surfaces while wiping, handling objects in damp conditions, and lifting a 2.5 kg payload (Supplementary Video 8 and Fig. 4c). In a rod-insertion task, the neuroprosthetic hand demonstrates consistent and stable pinching of slender rods of varying diameters (4, 6, 8 mm, each of *n* = 3 rods) and inserts them all into corresponding holes (Supplementary Video 9). Furthermore, Supplementary Video 10 and Fig. 4d demonstrate the subject continuously and stably grasping 20 distinct objects spanning diverse shapes, dimensions, weights, and textures. With the sEMG signals and decoded commands displayed alongside, the process from user intention to hand grasps is captured, while the hand’s compliance manages numerous uncertain physical interactions.

Supplementary Video 11 and Fig. 4e demonstrate the restored grasping capabilities of three unilateral amputee subjects in daily activities. The neuroprosthetic hand assists them in completing complex tasks that are typically challenging with only one healthy hand. For instance, it gently envelops fragile items (e.g., tofu) for safe transfer during cooking preparation; adaptively grasps both rigid and deformable objects during coffee making, thereby freeing the healthy hand for intricate tasks; and maintains a secure hold on nails under continuous hammering, demonstrating inherent impact resilience. Furthermore, its delicate pinch enhances bimanual coordination, as when an elderly user holds a pill bottle, allowing the healthy hand to open the bottle and access the pills. During vehicle operation, it maintains a stable grip on handlebars or steering wheels, freeing the healthy hand for brief actions like signaling or waving.

Moreover, our neuroprosthetic hand demonstrates remarkable advantages in enhancing amputees’ participation in typically dynamic social activities that require continuous adaptation to uncertainty. For instance, one female subject uses the neuroprosthetic hand to create a hairstyle for her daughter (Supplementary Video 12 and Fig. 4f). First, the soft fingers gather and align the hair into a strand by grasping, releasing, and re-grasping. No hair entanglement occurs due to the seamless soft covering of both fingers and the palm. Then, the fingers weave through the hair under guidance and mildly pull the strand, allowing the healthy hand to form a ponytail with an elastic loop. Finally, the fingers gently grasp and guide the hair to complete the bun. This inherently compliant interaction alleviates the typical anxiety associated with conventional prostheses, thereby facilitating intimate mother-daughter bonding. Overall, all amputee subjects benefit greatly from the neuroprosthetic hand’s high dexterity and compliance, from restoring daily grasping functions to performing complex tasks requiring bimanual coordination.

### Manipulation capability

**Fig. 5:**
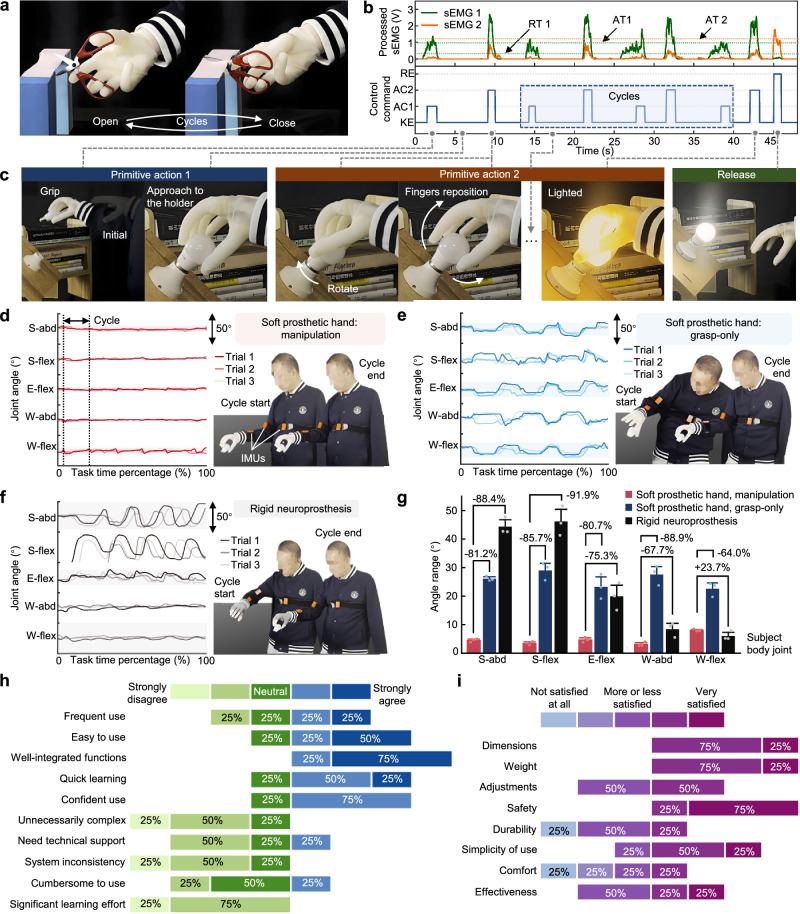
Manipulation capability and user-centered advantages. **a** Demonstration of an amputee subject using the neuroprosthetic hand to cyclically manipulate scissors for paper-cutting. **b** Time-varying sEMG signals and decoded control commands for a bulb installation task. **c** Multi-step manipulation demonstration for the bulb installation task. The subject picks up the bulb, secures it into the holder through cyclic screwing until the bulb is lit, and finally releases the hand. **d**–**f** Comparison of body compensatory movements in an amputee when wearing different neuroprosthetic hands during bulb installation tasks. Body compensatory movements are quantified through five key joint angles (*n* = 3 trials), monitored by three inertial measurement units (IMUs). Left panels display time-varying joint angles, and right panels show the amputee’s representative body postures at the initiation and completion of a screwing cycle. S-abd, shoulder abduction. S-flex, shoulder flexion. E-flex, elbow flexion. W-abd, waist abduction. W-flex, waist flexion. The values of S-flex > 50° are not displayed. **d** The amputee completes the task using the neuroprosthetic hand through two primitive motions. **e** The amputee completes the task using solely the grasping function of the neuroprosthetic hand. **f** The amputee completes the task using a commercial rigid neuroprosthetic hand. **g** Comparison of the angle range of five body joints for one subject. For each joint, the angle range is calculated as the mean range (maximum – minimum) across measurements (*n* = 3; error bar: s.d.). The results show the neuroprosthetic hand’s advantages in mitigating amputee unnatural compensatory movements. **h** Diverging bar charts of SUS user ratings. **i** Diverging bar charts of QUEST user ratings.

Manipulation is crucial yet unachievable in current prostheses (Supplementary Table 3). We explore the neuroprosthetic hand’s potential to address this challenge. We show (Supplementary Video 13 and Fig. 5a) that the neuroprosthetic hand can perform manipulation tasks, including consecutive scissor cuts, unlike single-snip^33^ demonstrations. Furthermore, Supplementary Video 14 and Supplementary Fig. 21 demonstrate that the neuroprosthetic hand can pick up and retain one object while simultaneously picking up a second object, and then sequentially place both objects. This continuous operation represents a form of manipulation that differs from conventional prostheses, which require releasing an object before picking up a new one. Supplementary Video 15 and Fig. 5b-c shows that the prosthesis can pick up, insert, and gradually screw in a bulb until it is lit (*n* = 5 trials, 100% success). This manipulation task requires at least two primitive actions: grips the bulb to establish contact, and rotates the gripped bulb before repositioning the fingers to release contact and prevent the bulb back-turn. Notably, the bulb-screwing process combines an active rotational torque with finger compliance to dynamically counterbalance the torque and manage uncertainties.

Using the bulb installation task as a case study, we examine how manipulation capabilities affect the kinematics of amputee bodies (Supplementary Video 16). Figure 5d–f compare three strategies: the manipulation and grasp-only modes of the neuroprosthetic hand, benchmarked against a rigid prosthesis. Body kinematics are quantified by five key joint angles (waist, shoulder, and elbow; see Supplementary Text 3 and Supplementary Fig. 22 for details) using inertial measurement units (IMUs). Figure 5g shows a substantially smaller total angular displacement across five joints for the manipulation strategy (24.03°) compared to the grasp-only strategy (123.05°) and rigid prosthesis (120.35°). A detailed analysis confirms the advantages of the manipulation strategy over the grasp-only strategy: decreasing shoulder abduction (81.19%), shoulder flexion (85.73%), elbow flexion (80.74%), waist abduction (88.94%), and waist flexion (64.01%). Reductions in shoulder abduction (88.42%) and flexion (91.92%) are amplified when compared to the rigid prosthesis. Besides, the task completion times across three strategies are comparable: manipulation, 8.5 ± 1.9 s; grasp-only, 8.7 ± 0.6 s; rigid prosthesis, 8.3 ± 0.4 s (mean ± s.d., *n* = 5 trials). This provides experimental evidence that our neuroprosthetic hand’s manipulation capability may mitigate compensatory body movements.

### User feedback evaluation

We next evaluate the feedback from all four amputee subjects with two surveys: System Usability Scale (SUS)^53^ and modified Quebec User Evaluation of Satisfaction with Assistive Technology (QUEST)^54^. Detailed results from each subject’s survey are provided in Supplementary Tables 16, 17. The overall average SUS score is 70.3 ± 15.1 (mean ± s.d., *n* = 4 subjects) on a 100-point scale, exceeding the acceptability threshold of 68 (Fig. 5h). Subjects appreciate the prosthetic hand’s integrated functions, ease of use, and quick learnability. While wearability presents challenges, subjects express neutral or positive about frequent use. QUEST results indicate satisfaction with key features, including weight, safety, and ease of adjustment (Fig. 5i). We use the NASA-TLX questionnaire^55^ to rate the subjective workload of our prosthetic hand for the subject when performing the manipulation tasks. The results (Supplementary Table 18) show low physical demand and moderate mental demand.

## Discussion

This work presents a new class of soft-robotic neuroprosthetic hand with high dexterity, overcoming the long-standing limitation of basic grasping (Fig. 1c). Coordinated through a robust two-channel myoelectric control interface, our neuroprosthetic hand translates the amputee user’s neural intent into dexterous motions, enabling versatile and adaptive grasping, near-natural hand function like intricate hair-braiding interactions, and delicate manipulation.

The high dexterity of our neuroprosthetic hand enables a design evolution (e.g., incorporating active lateral motion) for more specialized manipulation needs. Its force output suffices for a wide range of daily activities, while scenarios involving extreme loads call for enhanced maximum force capacity (Supplementary Table 2). Achieving this without compromising wearability and comfort remains a development goal; variable‑stiffness strategies^56^ offer a promising pathway by enabling on‑demand force modulation. Our neuroprosthetic hand demonstrates validated manipulation capability (Supplementary Table 3), which exhibits a degree of scalability without the need for reprogramming, except when handling objects with significant variations in size and shape. Its potential to reduce non‑natural body compensation, as shown in the bulb‑installation case study, offers a path toward more natural prosthetic use. In the future, advanced neural control algorithms^23,48^, e-skins^28^, haptic feedback^57^, and an active wrist shall be integrated to enable closed-loop sensorimotor control for more delicate manipulation tasks, followed by large‑scale trials involving a broader population of upper‑limb amputees.

## Methods

### Modeling of soft finger segments

We develop an analytical model of the soft finger segment, revealing the correlation between its bending angle and geometric structural parameters (detailed in Supplementary Text 1). We next model and analyze the movement of the soft finger segment in the ABAQUS (Dassault Systèmes). The structure of the finger segment is simplified as rectangular cavities with a wall thickness of 2 mm. The initial end surfaces of the finger segment are fixed as boundary conditions. The silicone elastomer is modeled as an incompressible Yeoh material to capture its hyperelastic behavior, with modeling parameters of C_10_ = 0.053 and C_20_ = 0.0015. The fiber reinforcement is simplified as a lattice layer with 0.36-Poisson’s ratio. The Quadratic 10-node modified tetrahedral elements (C3D10MH) are employed for the mesh. A quasi-static nonlinear analysis is performed by applying pressure to the inner surfaces of the finger segment, where the pressure range is set to 0–200 kPa. The finger segment length is chosen as the variable (20, 30, 40, 50 and 60 mm) for numerical analysis for the above pressure range. A larger bending angle can be achieved by increasing the finger segment length. Meanwhile, the bending angle per unit length exhibits a positive correlation with finger segment length up to 40 mm, after which it plateaus to a constant value. Accommodating anthropomorphic proportions and motion range requirements for human-like finger joints, 50 mm and 60 mm lengths are respectively chosen for the proximal segment (mimicking the human’s metacarpophalangeal joint) and distal finger segment (mimicking the synergistic motion of the human’s proximal and distal interphalangeal joints).

### Fabrication and assembly of the hand mechanism

The soft fingers are fabricated based on a multi-material, multi-step molding process. All molds are 3D-printed using selective laser sintering (SLS) with resin (Imagine 8000, SOMOS). The finger’s inner chamber is made of silicone elastomer Dragon Skin 20 (Smooth-On, *E* = 338 kPa under 100% strain). To acquire liquid Dragon Skin 20, its components (Parts A and B) are mixed 1:1 by mass using a planetary mixer (2500 rpm for 30 s; MZ-8, THINKY). The mixed Dragon Skin 20 is poured into assembled two-part molds, treated with vacuum defoaming, and cured at 25° for 7 h to obtain the inner chamber. The air tightness of finger segments requires separate sealing for independent actuation. Before sealing, all the components are pretreated with the surface treatment agent (Model 770, Valigoo). Silicone tube A (2.4 mm outer diameter) is inserted with silicone adhesive (Sil-poxy, Smooth On) into the tube channel between finger segments, followed by adhering the distal endcap to the inner chamber. Proximal segment sealing involves inserting tube-A and tube-B (2.4 mm outer diameter) into the proximal endcap channels with Sil-Poxy adhesive, then securing the proximal endcap to the inner chamber. Mechanical tying reinforces both endcaps for enhanced sealing. The strain-limiting layer made of polyethylene (*E* = 2 GPa, 0.2 mm thickness) is attached to the inner chamber’s bottom using cyanoacrylate adhesive (Model 502, Valigoo). Another 0.8 mm diameter polyethylene thread (Braided 4, YunShangPiao) is wound around the inner chamber, which is then assembled with another three-part mold to generate the finger skin. The skin material is a silicone elastomer: Ecoflex 00-30 (Smooth-On, *E* = 69 kPa under 100% strain). Its liquid components (Parts A and B) are mixed 1:1 by mass (3000 rpm for 60 s), followed by being injected into assembled molds, treated with vacuum defoaming, and cured at 25° for 6 hours to obtain the soft finger.

The rigid linkages of the palm joint are 3D-printed using SLS with nylon (7100Pro, Wenext). These linkages are compactly assembled with a micro servomotor (HV75, MKS) through bearings and rotating shafts. The palm joint is connected to the thumb using Sil-Poxy adhesive to form the thumb-palm composite. The palm skin is fabricated by applying a surface coating of the silicone elastomer Ecoflex 00-35 FAST (Smooth-On, *E* = 69 kPa under 100% strain). The surface is created using a hollow mold derived from a detailed human hand model, which is 3D-printed with a PolyJet printer (J750, Stratasys) using transparent resin (VeroClear, Stratasys). Components of this silicone elastomer are mixed 1:1 by mass using an injection system (MPD, SCME). The mixed silicone elastomer is coated onto the inner surface of the hollow mold and cured in about 2.5 minutes. Soft fingers and the palm-thumb composite are assembled onto the pre-designed mounting slots on the palm skeleton using both adhesive and threaded connections. Soft pads made of silicone elastomer Ecoflex 00-30 are attached to the palm surface with adhesive. Finally, the palm skin is fitted onto the palmar skeleton to complete the assembly of the hand mechanism.

### Performance measurements of the hand mechanism

To measure the movement of an individual finger, five reflective markers (3 mm diameter) are attached to the finger. Under pneumatic actuation, the trajectories of the markers along the finger’s bending motion are captured by a visual tracking system equipped with 4 cameras (Motive, OptiTrack). The actuation pressures for the finger segments are controlled via a data acquisition board (DS1103, dSPACE), by which the pneumatic actuation signals are converted to voltage signals to drive the pressure regulation modules. The pressure regulation modules consist of electric proportional regulators (MM1MBHEEN, Proportion-Air) and pressure sensors (Model 528, HUBA) and are used to proportionally regulate the outlet pressures according to the voltage signals. The actuation pressure for the proximal and distal finger segments is set to range from 0 kPa to 200 kPa (in 5 kPa intervals). By applying sweeps of actuation pressures to two finger segments, the finger workspace is generated based on the trajectory of marker 5 during the entire process. The finger is further actuated in three basic modes as well: only the proximal segment is actuated (termed proximal actuation, PA), only the distal segment is actuated (termed distal actuation, DA), and both segments share the same pressure (termed uniform actuation, UA). The trajectory data of markers 2, 3, 4 and 5 are used to calculate the bending angles of the different segments, denoted as *α* and *β*, and the entire finger in all three actuation modes. In the durability test, the soft finger undergoes a cyclic pressure actuation protocol featuring a 6 s cycle duration with a peak pressure of 100 kPa and equal inflation/deflation rates of 50 kPa/s for 10,000 times. The visual tracking system (OptiTrack) captures the trajectories of all the 5 markers on the finger, and the relationship between actuating pressure and bending angles is calculated. To measure the workspace of the hand mechanism, the trajectories of the markers on all fingertips are captured simultaneously. The actuation pressures of the fingers are set as 0–200 kPa in 5 kPa intervals. The proximal and distal segments of each finger share the same pressure. The actuation signal of the servomotor in the palm joint is set to range from 0° to 60° (in 5° intervals).

For the finger force test, the finger base is fixed, and a force sensor (LSB200, Futek) is mounted vertically below the fingertip to record the force in all three actuation modes. Actuation pressures are set as 0–200 kPa in 10 kPa intervals. For the force test of the hand mechanism, a universal testing machine (68SC-2, Instron) is used. The hand mechanism is fully actuated (with 200-kPa pressure in all segments) to firmly grasp a 3D-printed cylinder (55 mm diameter) connected to the force sensor of the universal testing machine. During the test, the universal testing machine moves the cylinder in one direction (at 5 mm/s) to pull it out of grasping, and the corresponding force curve is recorded. This test is conducted in both vertical and horizontal directions. Similarly, a 3D-printed ball (60 mm diameter) is used as the grasping object to test the pullout force of the hand mechanism. This test is conducted in six directions (palmar surface outward; 0°, 45°, 90°, 135°, 180° relative to the direction vertical to the middle finger longitudinal direction in the palmar surface). All tests are performed with *n* = 3 measurements for each direction.

### Control implementation and training protocol

Prior to functional evaluations, a standardized training protocol for amputee subjects to control the neuroprosthetic hand is implemented. The protocol consists of three sequential phases: (1) basic command training, where the amputee repeatedly practices the ‘Grasp’ and ‘Release’ commands until achieving stable performance; (2) speed modulation training, where the amputee practices the ‘Slow Active (SA)’, ‘Fast Active (FA)’, and ‘Release (RL)’ commands in sequence until achieving stable performance; and (3) mode transition training, where the amputee repeatedly practices the ‘Switch (SW)’ command for transitioning between different functional modes until achieving stable performance. The entire training protocol is completed within 10 min.

### Evaluation of user control stability

Four amputee subjects participate the test. Testing sessions are conducted at four time points: 09:00, 13:00, 17:00, and 21:00. During each test session, participants, while wearing a neuroprosthetic device, are instructed to perform specific movements by contracting the corresponding muscles in response to verbal cues. Each session comprised fifty commands spanning five categories, including SW, RL, AC1, AC2, and KE, which are arranged in a random order. During the experiment, upon receiving a verbal cue, the amputee subject replicates the instructed movement by contracting the corresponding muscles. We record both the instructed command and the actual output of the subject.

### Electrochemical impedance spectroscopy test

An amputee (subject 1) participates in the impedance testing with his residual forearm’s skin interfacing the sEMG sensor via the arm socket. We attach the three electrodes of an sEMG sensor (reference, counter, and working electrodes) using three strips made of metal-based conductive fabrics (Model 2010, 3 J). Connected through these fabric strips, an electrochemical workstation (AutoLab, Metrohm) measures the skin impedance across a frequency range from 1 Hz to 10⁵ Hz. Measurements are performed at 4 h intervals within 12 h, all employing 10 mV (peak-to-peak amplitude) sinusoidal signals.

### Standardized grasping tests setups

In the BBT, two boxes are placed adjacently, with a baffle plate between them, and scattered cubic blocks (25.4 mm edge length, 8.5 g weight) are placed in one of the boxes. The amputee is instructed to use the neuroprosthetic hand to grasp the blocks from one box and transfer them to another box as quickly as possible. The amputee can choose their preferred hand gesture and body posture (sitting or standing) during the test. We use a smartphone (Note 13Pro, Redmi) to set a one-minute timer and the number of transferred blocks is counted. Multiple blocks transferred simultaneously count as one transfer. We evaluate BBT on amputees (*n* = 4, including 3 males and 1 female, ages 38–70 years). Prior to testing, the amputee is given 5 min to practice. The test is repeated *n* = 3 times per amputee subject.

In SHAP, the following 12 tasks are performed: S1, light power; S2, heavy power; S3, light sphere; S4, heavy sphere; S5, light tip; S6, heavy tip; S7, light extension; S8, heavy extension; S9, light tripod; S10, heavy tripod; S11, light lateral; S12, heavy lateral. In each task, the amputee is instructed to use a specified hand gesture of the neuroprosthetic hand to move the designated object from the start point to a given place as quickly as possible. The completion time is self‑recorded by the subject using a push‑button timer. The objects used in SHAP vary in shape, weight, and size. We evaluate SHAP on amputees (*n* = 4, including 3 males and 1 female, ages 38–70 years). Prior to testing each task, the amputee is given 5 min to practice. To reduce fatigue, the amputee is asked to rest for 1 min between every task. Each task is repeated *n* = 3 times per amputee subject.

### Human participants

All experiments were conducted in accordance with the declaration of Helsinki and approved by the Institutional Review Board for Human Research Protections of Shanghai Jiao Tong University (E20250927C). The amputees who participated in this study are recruited from the general population. The participants did not have any previous neuromuscular disorders and were informed about the experimental procedure and signed the informed consent forms before participation. The authors affirm that human research participants provided written informed consent for the publication of the images.

### Ethics

Every experiment involving animals, human participants, or clinical samples have been carried out following a protocol approved by an ethical commission. Each participant gave informed written consent.

### Reporting summary

Further information on research design is available in the Nature Portfolio Reporting Summary linked to this article.

## Supplementary information

Supplementary Information

Description of Additional Supplementary Information

Supplementary Video 1

Supplementary Video 2

Supplementary Video 3

Supplementary Video 4

Supplementary Video 5

Supplementary Video 6

Supplementary Video 7

Supplementary Video 8

Supplementary Video 9

Supplementary Video 10

Supplementary Video 11

Supplementary Video 12

Supplementary Video 13

Supplementary Video 14

Supplementary Video 15

Supplementary Video 16

Reporting Summary

Transparent Peer Review file

## Source data

Source Data

## Data Availability

All data supporting the findings of this study are available within the article and its supplementary files. Any additional requests for information can be directed to, and will be fulfilled by, the corresponding authors. Source data are provided in this paper.
