# Supplementary material for: A high-dexterity soft neuroprosthetic hand for daily activities
Source: Nat Commun. 2026 Jul 4;17:8320. doi: 10.1038/s41467-026-75105-6 (PMC13470212; doi:10.1038/s41467-026-75105-6)
Supplement: Supplementary file 2 [file 41467_2026_75105_MOESM1_ESM.pdf]

Supplementary Information

A high-dexterity soft neuroprosthetic hand for daily activities

Ningbin Zhang[1#*], Xinyu Yang[1#], Zheng Zong[1], Yang Yu[2,3], Yi Zhao[1], Rong Bian[1], Chengru Jiang[1], Fengjie Shen[1], Xiangyang Zhu[1,2,3*], Guoying Gu[1,2*]

[1]State Key Laboratory of Mechanical System and Vibration, School of Mechanical Engineering, Shanghai Jiao Tong University, Shanghai 200240, China.
[2]Shanghai Key Laboratory of Intelligent Robotics, Shanghai Jiao Tong University, Shanghai 200240, China.
[3]Meta Robotics Institute, Shanghai Jiao Tong University, Shanghai 200240, China.

[#]These authors contributed equally to this work.
[*]Corresponding author. Email: guguoying@sjtu.edu.cn, mexyzhu@sjtu.edu.cn, zhangnb@sjtu.edu.cn

The PDF file includes:

Supplementary Texts 1 to 3

Supplementary Figures 1 to 22

Supplementary Tables 1 to 18

Other Supplementary Information for this manuscript includes the following:

Supplementary Videos 1 to 16

Supplementary Text 1

Analytical modelling of finger segments

We develop an analytical model of the fiber-reinforced soft finger segment (**Supplementary Figure 1a**). In the finger segment, the elastomeric chamber is approximated as a rectangular prism. Its thickness, inner height, and inner width are defined as *t*, *h*, and *w*, respectively. The elastomeric chamber is modelled as an incompressible Neo-Hookean material with an initial shear modulus of μ. The polyethylene sheet is modeled as a flexible but inextensible layer. Initially, the soft finger segment exhibits an axial length of *L*. Under applied pneumatic pressure *P*, the axial elongation of the finger segment (*y*-direction) can be expressed as:

$$\lambda_z\left(y\right)=\frac{y\left(\lambda_z\left(h\right)-1\right)}{h}+1, \tag{S1}$$

where $\lambda_z\left(h\right)=\lambda_z^h=l/L$ denotes the axial stretch of the upper surface, and *l* denotes the stretched length. The strain energy density of the elastomeric chamber, *W*, is given by

$$W=\frac{\mu\left(I_1-3\right)}{2}. \tag{S2}$$

The parameter I_1 is the first invariant of the deformation gradient **F**:

$$I_1=\mathrm{tr}\left(\mathbf{F}\mathbf{F}^{\mathrm{T}}\right)=\lambda_1^2+\lambda_2^2+\lambda_3^2. \tag{S3}$$

In equation S3, the deformation gradient **F** is expressed as:

$$\mathbf{F}=\begin{bmatrix}\lambda_1 & 0 & 0\\ 0 & \lambda_2 & 0\\ 0 & 0 & \lambda_3\end{bmatrix} \tag{S4}$$

where λ_1, λ_2, and λ_3 denote the radial, circumferential, and axial stretches, respectively. Material incompressibility imposes the constraint:

$$\lambda_1\cdot\lambda_2\cdot\lambda_3=1. \tag{S5}$$

The circumferential constraint from fibers suppresses elastomeric chamber's torsional deformation, allowing the circumferential strain to be neglected ($\lambda_2 = 1$). Thus, we have:

$$\lambda_1=\lambda_z^{-1}\left(y\right),\ \lambda_2=1,\ \lambda_3=\lambda_z\left(y\right). \tag{S6}$$

The deformation gradient **F** can be rewritten as:

$$\mathbf{F}=\begin{bmatrix}\lambda_z^{-1}\left(y\right) & 0 & 0\\ 0 & 1 & 0\\ 0 & 0 & \lambda_z\left(y\right)\end{bmatrix}. \tag{S7}$$

By combining equations S2, S3 and S7, we obtain:

$$W=\frac{\mu\left(\left(\lambda_z^{-1}(y)\right)^2+\left(\lambda_z(y)\right)^2+1-3\right)}{2}. \tag{S8}$$

The principal nominal stresses S_i (i = 1,2,3) of the elastomeric chamber can be calculated by

$$S_i=\frac{\partial W}{\partial \lambda_i}-\frac{\xi}{\lambda_i}, \tag{S9}$$

where ξ is the Lagrange multiplier. As the radial stress is negligible, we have:

$$S_1=\frac{\partial W}{\partial \lambda_1}-\frac{\xi}{\lambda_1}=\mu\lambda_1-\frac{\xi}{\lambda_1}=0. \tag{S10}$$

Thus, the Lagrange multiplier ξ can be expressed as a function of y, which is solved by

$$\xi(y)=\mu\lambda_1^2=\mu\lambda_z^{-2}(y). \tag{S11}$$

By combining equations S9 and S11, the circumferential stress is derived:

$$S_2(y)=\frac{\partial W}{\partial \lambda_2}-\frac{\xi(y)}{\lambda_2}=\mu\left(1-\lambda_z^{-2}(y)\right)=\mu-\mu\left[\frac{y}{h}\left(\lambda_z^h-1\right)+1\right]^{-2}. \tag{S12}$$

The axial stress S_3 is calculated by

$$S_3(y)=\frac{\partial W}{\partial \lambda_3}-\frac{\xi(y)}{\lambda_3}=\mu\left(\lambda_z(y)-\lambda_z^{-3}(y)\right)=\mu\left[\frac{y}{h}\left(\lambda_z^h-1\right)+1\right]-\mu\left[\frac{y}{h}\left(\lambda_z^h-1\right)+1\right]^{-3}. \tag{S13}$$

The relationship between elastomeric chamber's bending angle α and parameters (l, L, and h) is established by

$$\alpha=\frac{l-L}{h}. \tag{S14}$$

The elastomeric chamber's bending motion arises from the moment M_P induced by applied pressure P acting on the end cap. The relationship between P and α is derived by equating the moment M_C (calculated from internal stresses) to M_P:

$$M_C=M_P. \tag{S15}$$

The neutral axis of bending is along the polyethylene sheet layer, thus M_C is derived by

$$M_C=2\int_0^h S_3(y)ytdy+S_3(h)wht. \tag{S16}$$

The moment M_P is expressed as

$$M_P=\int_0^h Pwydy. \tag{S17}$$

Solving equations S14-S17 yields the P-α relationship (with λ_z^h as an intermediate variable):

$$\begin{cases}\dfrac{2t}{3w\left(\lambda_z^h-1\right)^2}\left[2\left(\lambda_z^h\right)^3-3\left(\lambda_z^h\right)^2+6\left(\lambda_z^h\right)^{-1}-3\left(\lambda_z^h\right)^{-2}-2\right]+\dfrac{2t}{h}\left[\left(\lambda_z^h\right)-\left(\lambda_z^h\right)^{-3}\right]=\dfrac{P}{\mu}\\ \alpha=\dfrac{(\lambda_z^h-1)L}{h}\end{cases}. \tag{S18}$$

Supplementary Text 2

Typical FSM-based control

Two sEMG channels (each with 2 states) generate $2^2 = 4$ combined states (i.e., “Execute current grasp mode”, “Release from current grasp mode”, “Switch to next grasp mode”, and “Rest”). This control strategy in principle allows cycling through all grasping modes that the hand mechanism can physically achieve, and the practical number of modes is tailored to the user’s preference, balancing functionality with the need to avoid an overly cumbersome cycling sequence. For any given grasp mode, the states of “Rest” and “Switch to next grasp mode” are reserved for global management, leaving only the “Execute current grasp mode” and “Release from current grasp mode” to control the grasp mode itself. This results in binary on/off grasps without further programmable modulation.

Supplementary Text 3

Measurement and calculation of postural compensation angles

We use inertial measurement units (IMUs) to measure the amputee subject's postural compensations when he uses different neuroprosthetic hands in the light bulb manipulation task. To this end, three IMUs (Awinda, Xsens) are used to measure the joint angles of the elbow, shoulder, and waist (**Supplementary Figure 22a-b**). Before measurements, all three IMUs are put in alignment with their z-axis parallelly pointing straight up and then uniformly calibrated (**Supplementary Figure 22c-d**), by which their coordinate frames are unified using data collection software (MT Manager 2022, Xsens). IMU-1 is attached to the amputee subject's chest with its x-axis in alignment with the central axis of the main body. IMU-2 and IMU-3 are respectively attached to the upper arm and forearm, with their x-axis in alignment with them (**Supplementary Figure 22e**). During the experiment, the data collection software records the pose of all the IMUs in the entire process in the form of Euler angles. From the recorded data, we obtain the coordination system information of all IMUs, by which the joint angle can be calculated. The x-axis of IMU-1 is used to generate the waist abduction and flexion angle by projection to calibrated coordinate planes. The information from IMU-1 and IMU-2 is used to generate the shoulder abduction and flexion angle by projecting the x-axis of IMU-2 to the coordinate planes of IMU-1. The x-axis information of IMU-2 and IMU-3 is used to calculate the elbow flexion angle (**Supplementary Figure 22f**). After the joint angle variations during the task are obtained, all the joints' compensation motion ranges are obtained by making a difference between the maximum and minimum joint angles (**Supplementary Figure 22g**). The calculation procedures are as follows:

$$ {}_{m}^{n}\mathbf{R} = \begin{pmatrix} {}_{m}^{n}\mathbf{x} & {}_{m}^{n}\mathbf{y} & {}_{m}^{n}\mathbf{z} \end{pmatrix} = \begin{bmatrix} {}_{m}^{n}a & {}_{m}^{n}d & {}_{m}^{n}g \\ {}_{m}^{n}b & {}_{m}^{n}e & {}_{m}^{n}h \\ {}_{m}^{n}c & {}_{m}^{n}f & {}_{m}^{n}i \end{bmatrix} \tag{S19} $$

$$ \begin{gathered} \theta_{wa} = \arctan\left(\frac{{}_{1}^{0}b}{{}_{1}^{0}c}\right), \theta_{wf} = \arctan\left(\frac{{}_{1}^{0}a}{{}_{1}^{0}c}\right), \theta_{sa} = \arctan\left(\frac{-{}_{2}^{1}b}{{}_{2}^{1}a}\right), \\ \theta_{sf} = \arctan\left(\frac{-{}_{2}^{1}c}{{}_{2}^{1}a}\right), \theta_{ef} = 180^{\circ} - \arctan\left(\frac{\overrightarrow{{}_{1}^{0}\boldsymbol{x}} \cdot \overrightarrow{{}_{2}^{0}\boldsymbol{x}}}{\left|\overrightarrow{{}_{1}^{0}\boldsymbol{x}}\right|\left|\overrightarrow{{}_{2}^{0}\boldsymbol{x}}\right|}\right) \end{gathered} \tag{S20} $$

Here, θ_{wa}, θ_{wf}, θ_{sa}, θ_{sf}, θ_{ef} stands for the joint angle of waist abduction, waist flexion, shoulder abduction, shoulder flexion and elbow flexion, respectively. ${}_{m}^{n}\mathbf{R} = \begin{pmatrix} {}_{m}^{n}\mathbf{x} & {}_{m}^{n}\mathbf{y} & {}_{m}^{n}\mathbf{z} \end{pmatrix}$ represents the *x, y, z* axes of the *m*th IMU's coordinate system expressed in the *n*th IMU's coordinate system, where ${}_{m}^{n}\mathbf{R}$ for all the IMUs can be obtained directly by measurement.

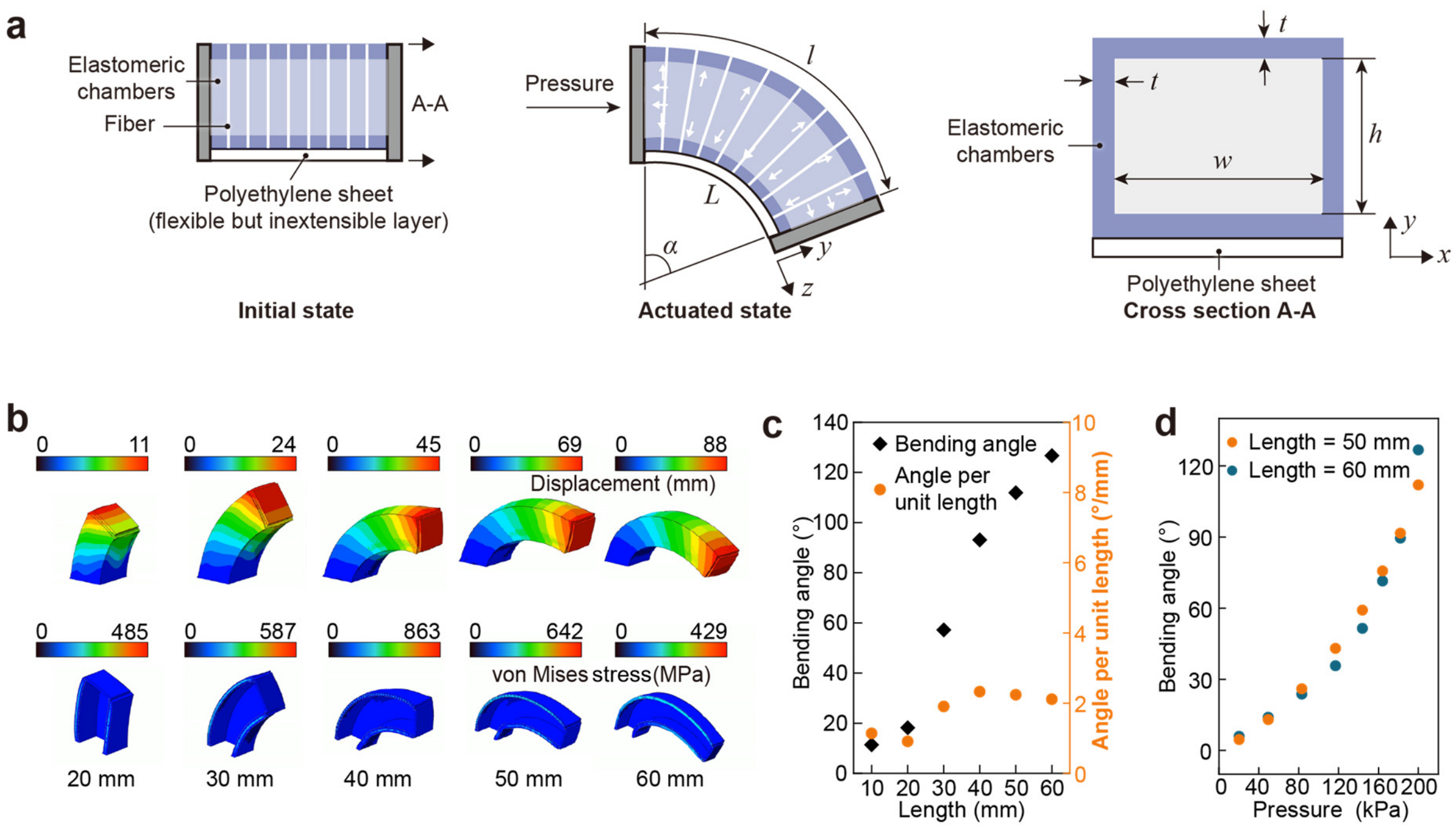

Supplementary Figure 1. Pneumatic soft bending actuator design. a, Working principle of a soft finger segment. Upon pneumatic actuation, the segment bends by an angle α due to the differential stretching between its upper and lower parts. An analytical model can be established involving several key structural parameters, which is detailed in Supplementary Text 1. L, initial length. l, stretched length. t, elastomeric chamber's thickness. h, its inner height. w, its inner width. **b**, Finite-element simulations on the tip displacements and von Mises strains for finger segments with different initial lengths. **c**, Bending angle and angle per unit length as functions of the segment's initial length. **d**, Bending angle as a function of the applied pressure for the chosen segments' initial lengths.

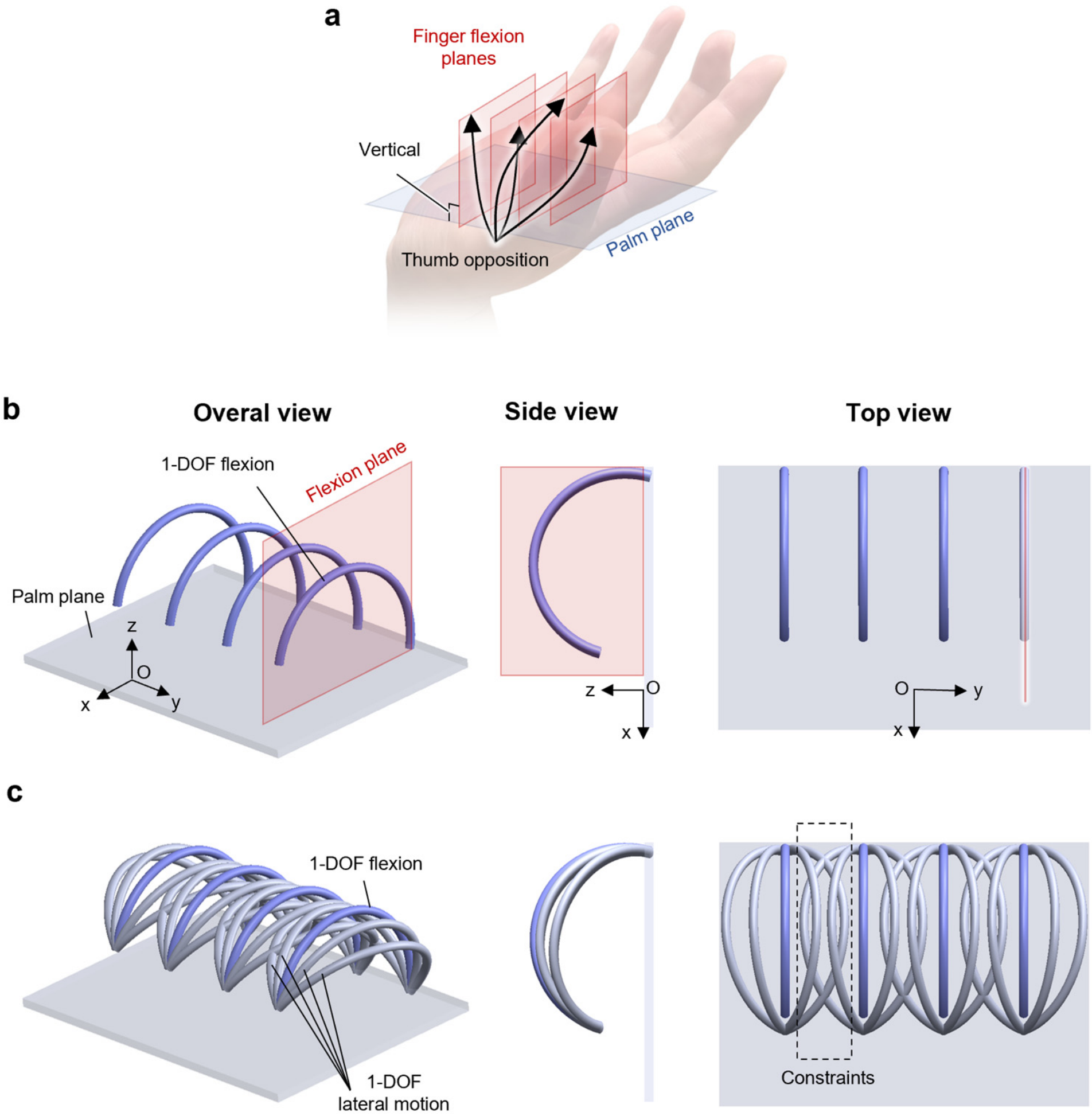

Supplementary Figure 2. Design prioritization among multiple DOFs. a, Schematic principle of finger flexion in the human hand. The four fingers flex in approximately parallel planes (flexion planes) nearly vertical to the palm. These motions coordinate with thumb movement, and their combined kinematics give rise to the primary function of thumb opposition. **b**, Schematic principle of a prosthetic hand with active 1-DOF finger flexion. The side view shows that motion in the flexion plane follows a curved trajectory, which results in a limited workspace within this plane. **c**, Schematic principle of active 2-DOF finger flexion and lateral motion. The side view and top view show that the workspace within the flexion plane remains limited, a constraint that is further compounded by physical interference from adjacent fingers. Yet, under external forces or actions from adjacent fingers and the opposable thumb, this very interference can induce soft fingers' passive lateral motion for adaptive grasp and manipulation. Therefore, finger lateral motion is not eliminated but achieved through a form of embodied intelligence.

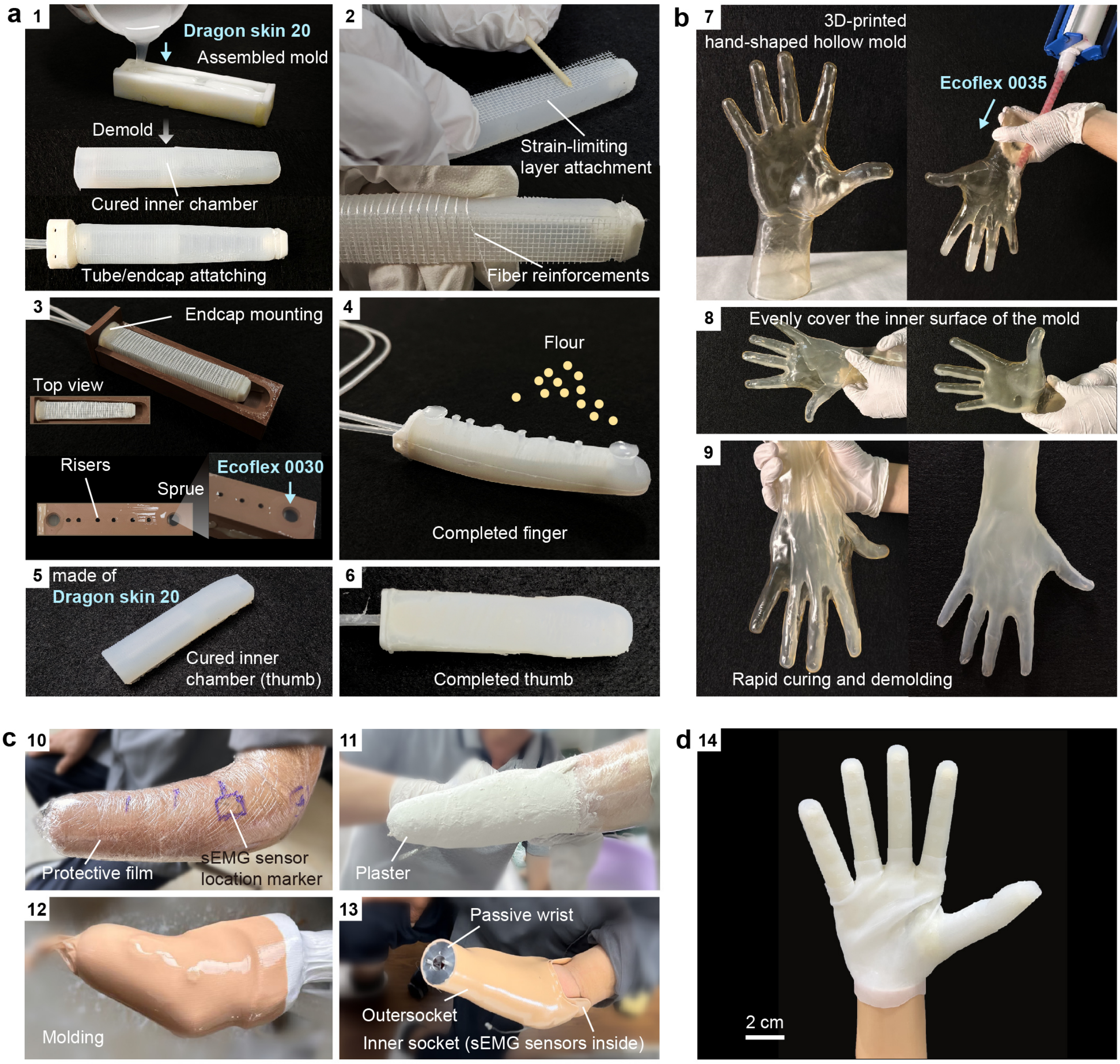

Supplementary Figure 3. Fabrication and assembly of the high-dexterity soft neuroprosthetic hand. a, Fabrication process of the soft finger/thumb. The liquid silicone is cast into the assembled mold to obtain the inner chamber. Tubes and endcaps are attached to the inner chamber, and the strain-limiting layer (polyethylene sheet) and fiber winding are applied to the inner chamber. Then, the inner chamber is assembled to an outer mold and recast for the finger skin. After demolding, excess silicone rubber is trimmed to obtain the soft finger/thumb, and a small amount of flour is applied to remove any residual stickiness on the silicone surface. **b**, Fabrication process of the palmar skin. The liquid silicone rubber is injected into the inner surface of a hollow hand mold. The mold's orientation is manually adjusted to ensure an even coating of silicone on the inner surface. After rapid curing for 5 minutes, the soft skin is obtained, and the digits are trimmed to form the palmar skin. **c**, Fabrication process of the prosthetic arm socket. The positions of active muscle groups on amputee subject's residual limb are identified and marked. The residual limb is wrapped with wet plaster bandages, which solidify to create a plaster mold for the inner socket. Two sEMG sensors are embedded in the molded inner socket at the marked muscle group locations. An outer socket is fabricated to mimic the intact forearm, with a passive wrist embedded in the distal end of the outer socket. **d**, Assembled hand mechanism. Pneumatic tubes and electrical wires route through the wrist to the arm socket.

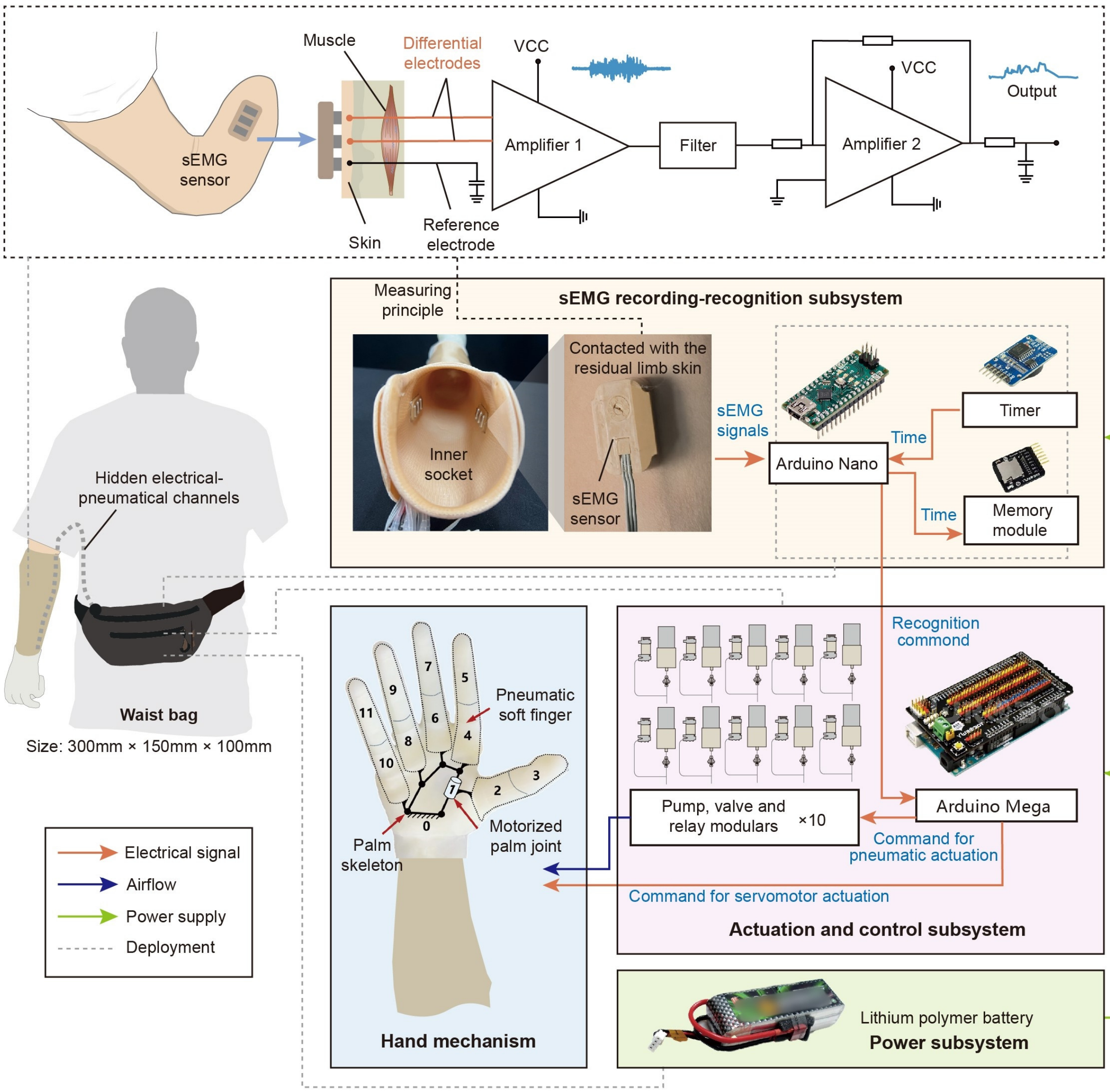

Supplementary Figure 4. Wearable actuation and control system. The system is integrated into a waist bag, which consists of the power subsystem, sEMG recording-recognition subsystem, and actuation-control subsystem. The power subsystem mainly includes a lithium polymer battery (1300-mAh capacity, 11.1-V voltage, ACE) and buck converters, which provide regulated electrical supplies. The sEMG recording-recognition subsystem comprises two sEMG sensors (1205, Danyang) for recording sEMG signals from the amputee's residual limb, a microprocessor (Nano, Arduino) for signal processing and recognition, a timing module (DS3231, Zave) for recording the system running time, and a data storage module (MicroSD Module, YwRobot). Raw signals from each sEMG sensor are processed through amplification and filtering. The actuation-control subsystem comprises a microprocessor (Mega, Arduino) for translating the recognized sEMG signal into the control commands of hand movements; these commands drive the motor, micro pumps (3.5-lpm flow rate, 185-kPa maximum pressure, Fspump) for providing air supply, and micro valves (2-way normally closed, x-valve, Parker) for offering rapid pneumatic control (about 20-ms response time). The micro pumps and valves are arranged in custom electrical circuits. All the electrical wires and pneumatic tubes are integrated into a flexible sleeve, which is hidden under clothing when the amputee uses the prosthetic hand.

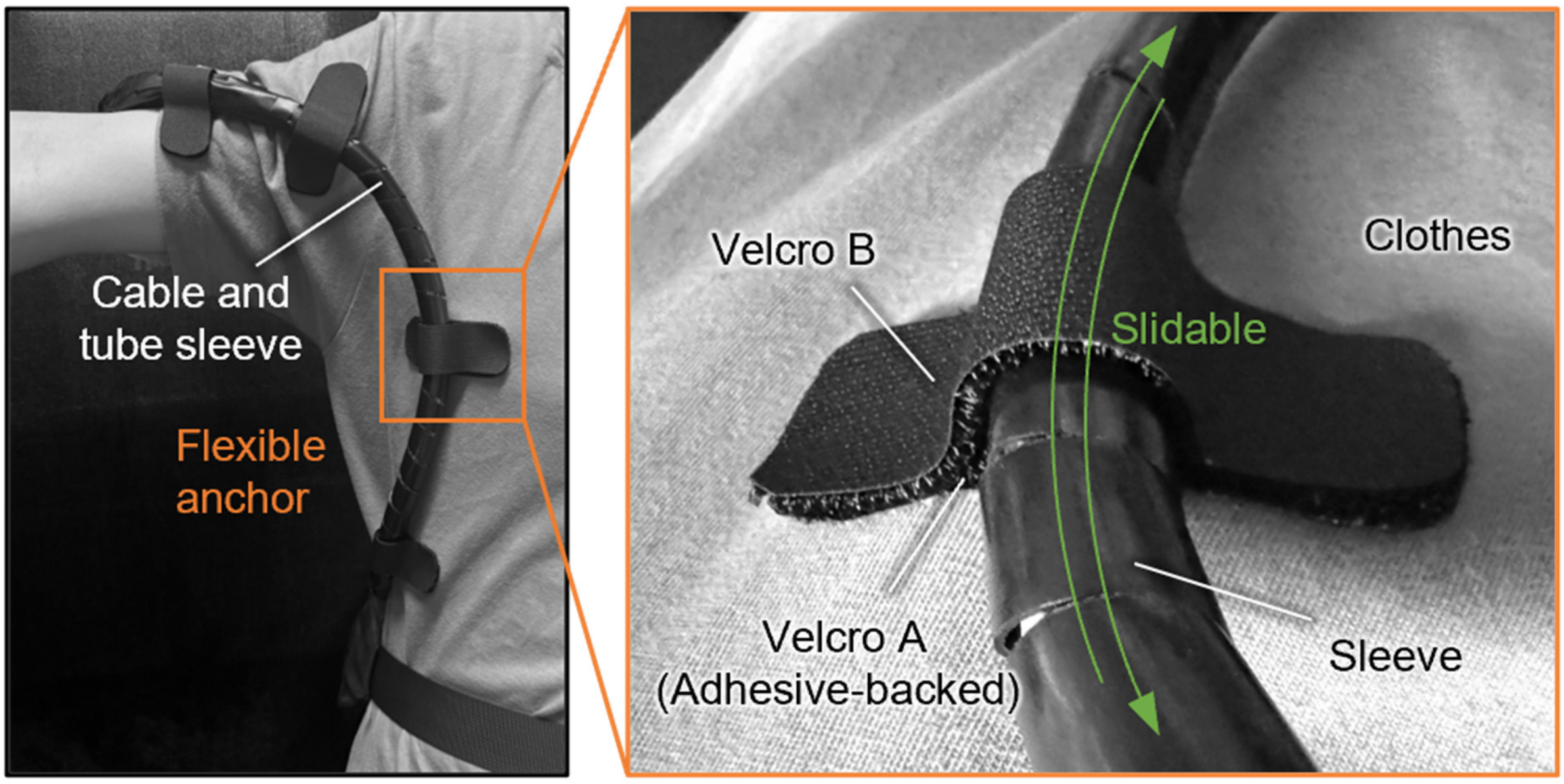

Supplementary Figure 5. Flexible Velcro-based anchors for cable and tube routing. These anchors are designed to be attached to the user's clothing, conforming to body movements for adaptable routing. Each anchor consists of two Velcro parts (A and B). Part A features an adhesive backing for secure attachment to fabrics and is shaped to preserve an "Ω"-shaped space. Part B physically attaches to Part A through a hook-and-loop interface, thereby enabling a secure connection that is also easily adjustable and detachable. The sleeve containing the electrical wires and pneumatic tubes is slidable within this "Ω"-shaped Velcro pair, thus minimizing interference during body movement and social interactions. Additionally, the bag is constructed from a lightweight nylon material to maintain a low profile, even when worn over thicker clothing.

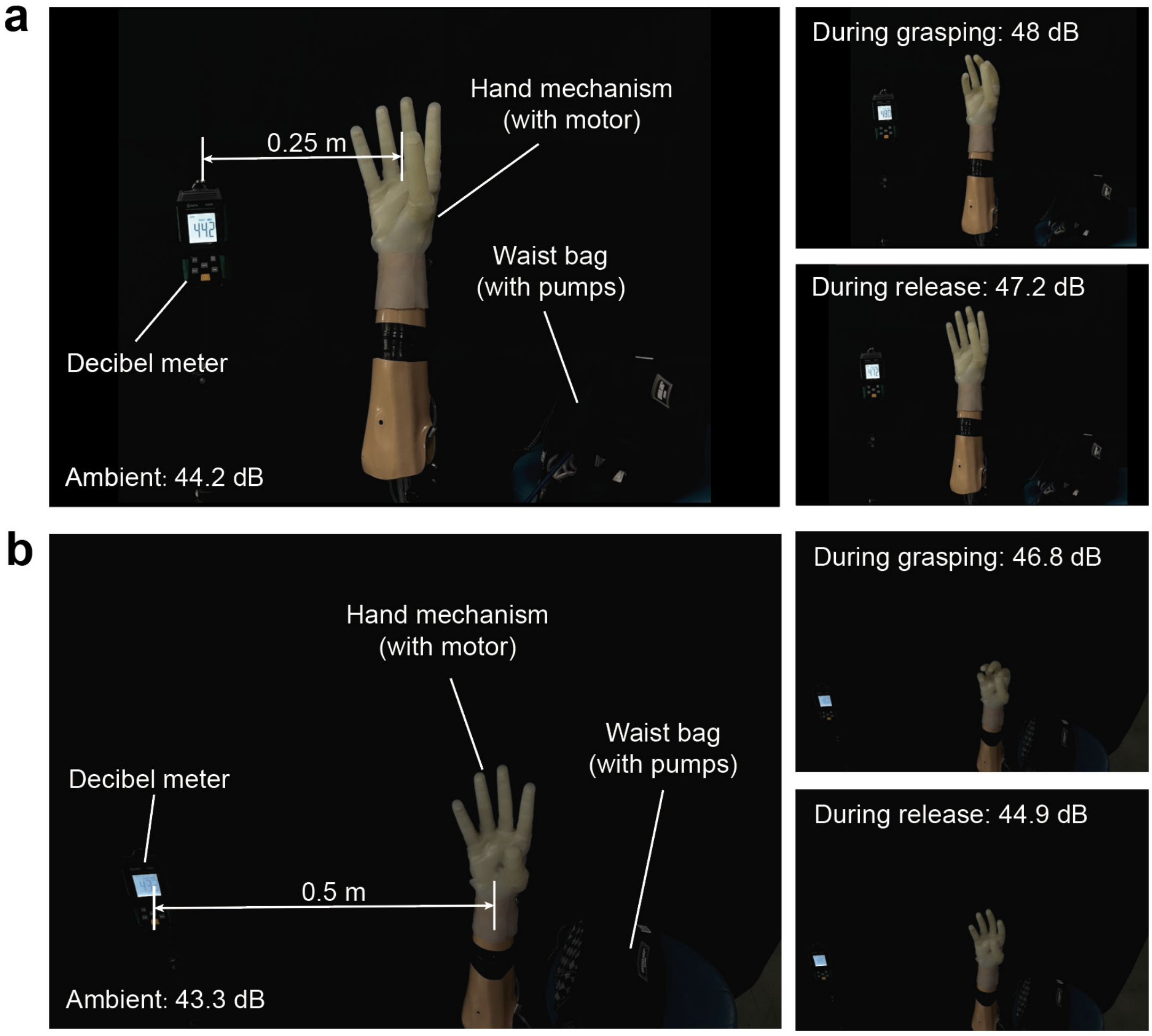

Supplementary Figure 6. Noise measurements. a, Setup for measurement at a distance of 0.25 m using a decibel meter. With the motor in the hand and pumps in the waist bag, the recorded levels are 44.2 dB (ambient), 48.0 dB (during grasp), and 47.2 dB (during release). **b**, Setup for measurement at a distance of 0.5 m using the same decibel meter. The recorded levels are 43.3 dB (ambient), 46.8 dB (during grasping), and 44.9 dB (during release).

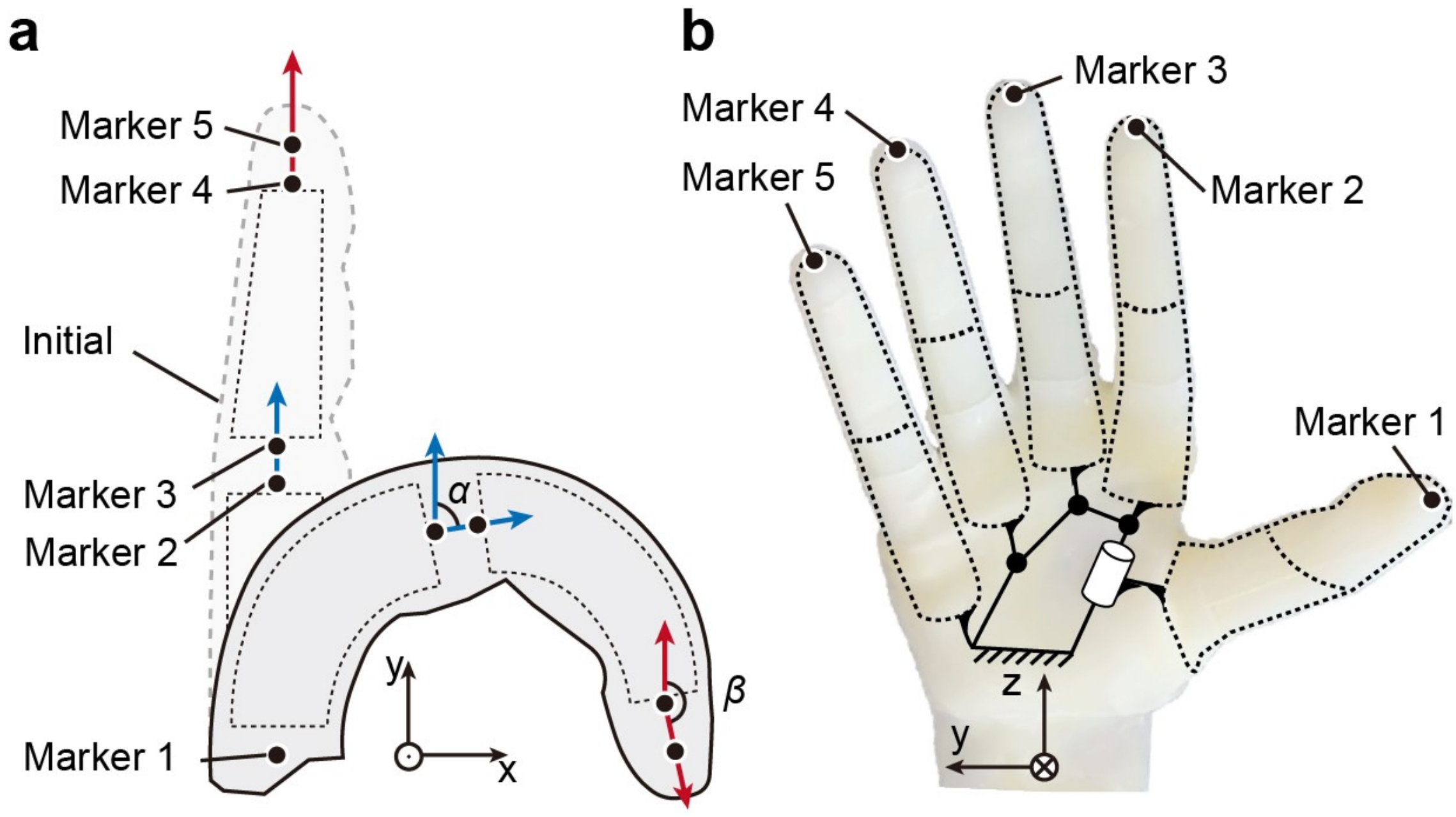

Supplementary Figure 7. Kinematic capability measurements. a, Workspace measurement of an individual finger or the thumb. Taking the marker layout of a finger as an example, the fingertip position and bending angles can be obtained. **b**, Workspace measurement of the hand mechanism.

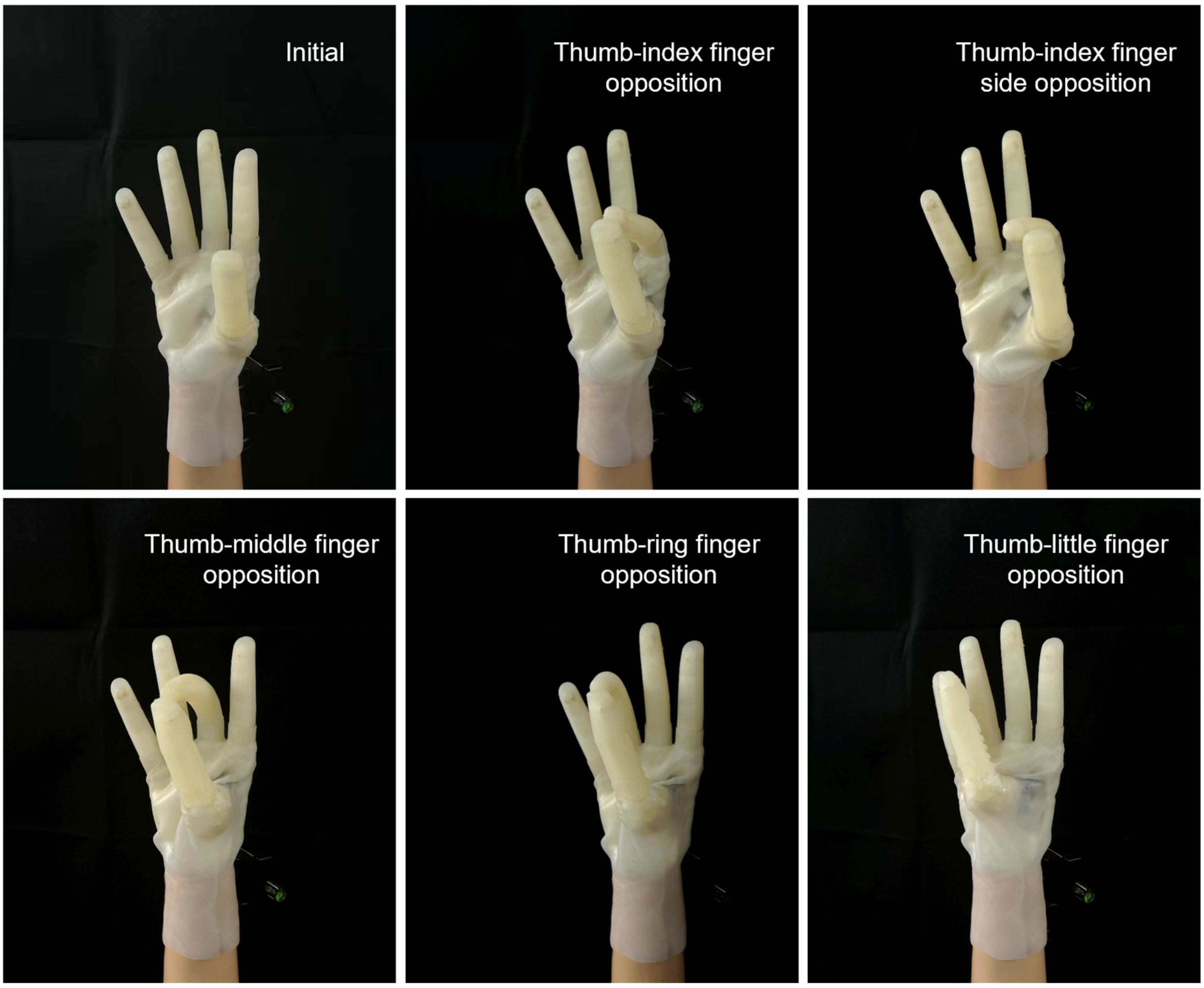

Supplementary Figure 8. Demonstration of thumb opposition with other fingers. The opposition is achieved by the coordinated motion of the thumb-palm composite and fingers.

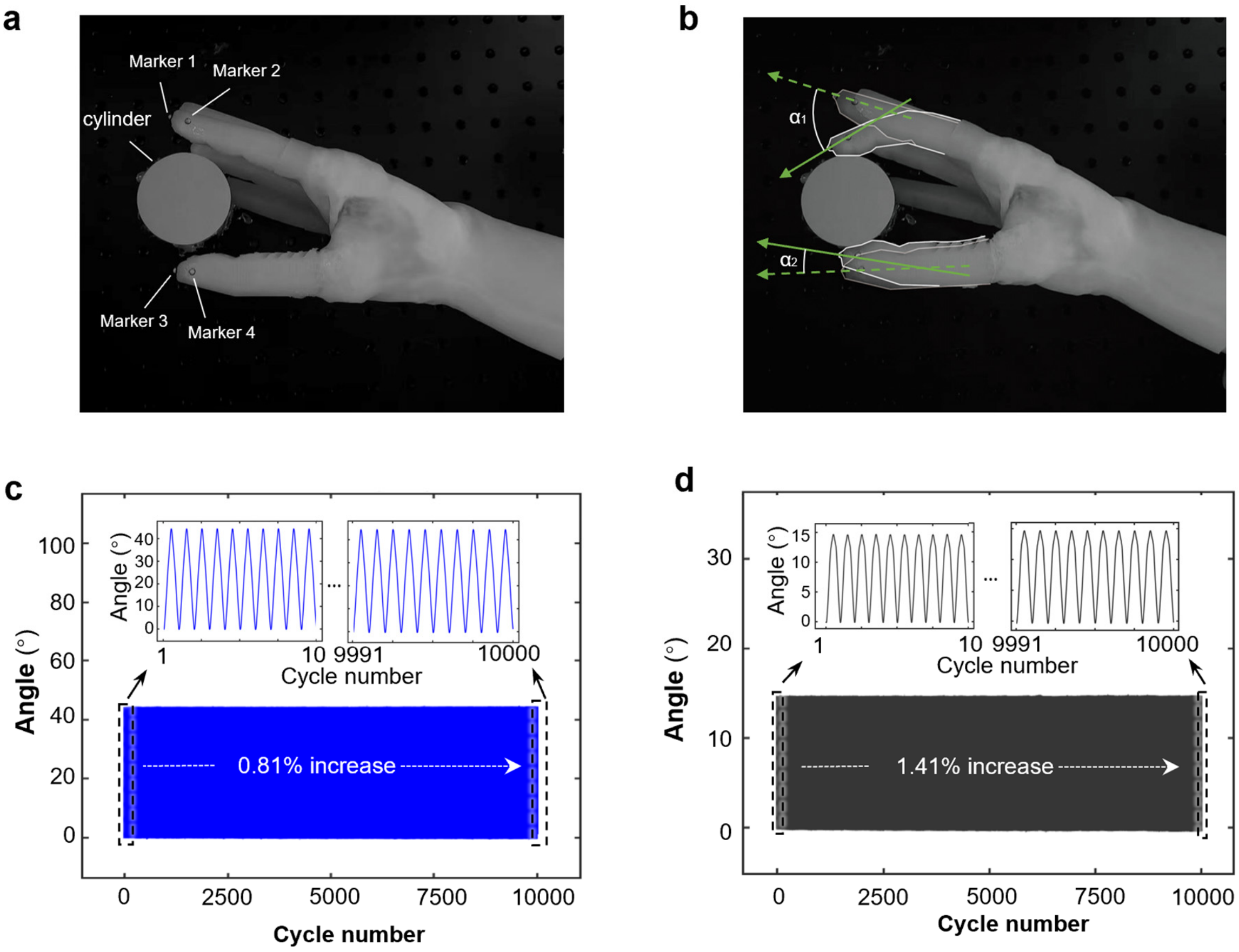

Supplementary Figure 9. Durability test with object contact. a, Description of the experimental setup. We mount the neuroprosthetic hand and a cylinder (diameter: 60 mm) on the test table. Markers 1 and 2 are attached to the index finger, while markers 3 and 4 are placed on the thumb. A pneumatic system applies periodic actuation pressure (0-100 kPa) at 2-second intervals to control the neuroprosthetic hand to grasp and release, with the actuation cycle repeated over 10,000 cycles. **b**, Schematic illustration of the grasping process with flexion angle annotations. The flexion angle of the index finger (α_1) and that of the thumb (α_2) are calculated based on the vectors formed by the paired markers. **c**, Variation in the finger flexion angle during 10,000 grasping cycles. The flexion angle of the index finger remains stable throughout the durability test, with no significant changes observed (only 0.81% increase). **d**, Variation in the thumb flexion angle during 10,000 grasping cycles. Similar to the index finger, the thumb maintains a stable flexion angle throughout the test (only 1.41% increase), confirming the long-term reliability.

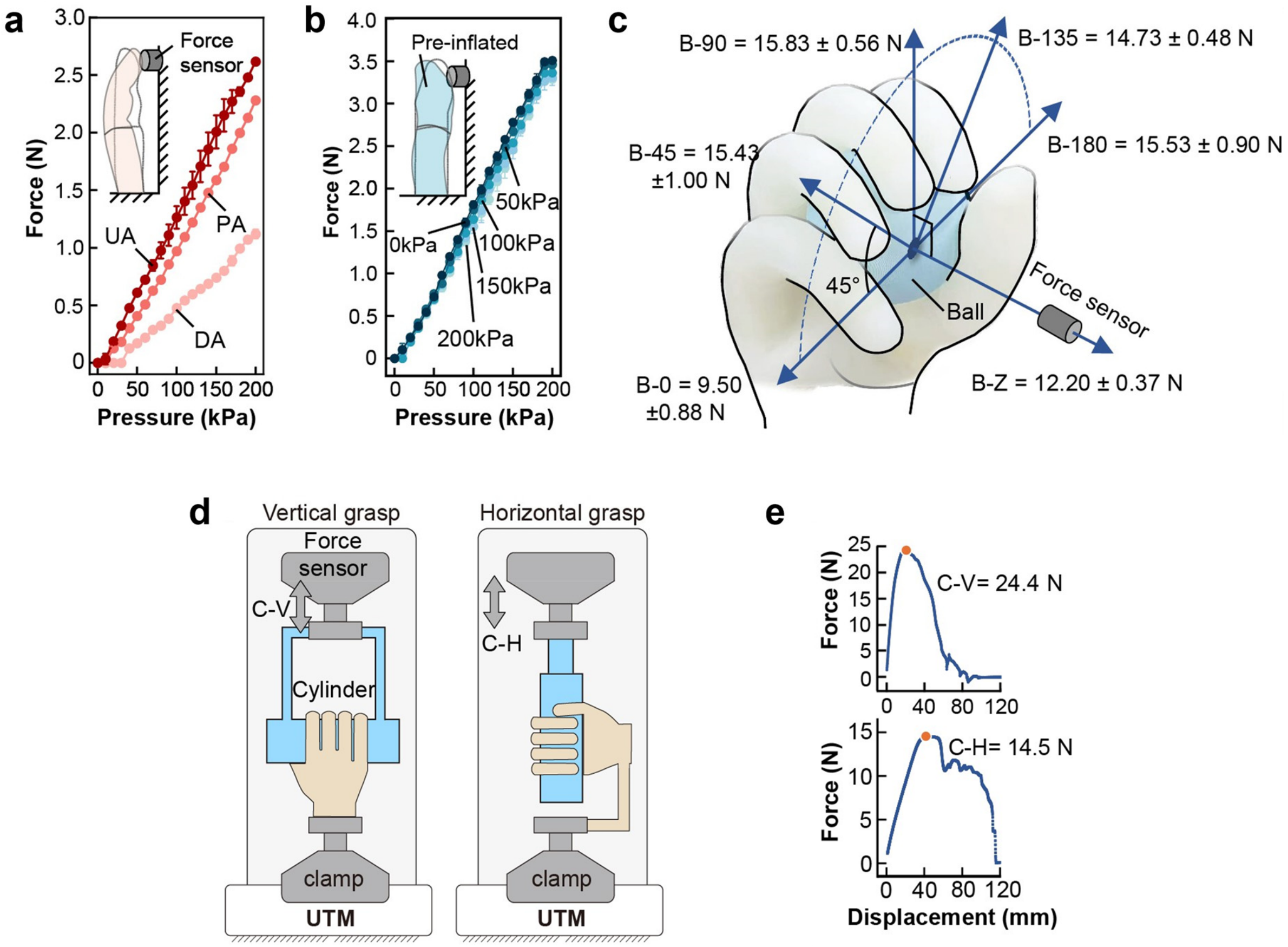

Supplementary Figure 10. Force capability measurements. a-b, Output finger/thumb forces as functions of the actuation pressure. The finger is driven in proximal actuation (PA) mode, distal actuation (DA) mode, and unified actuation (UA) mode. The thumb is actuated with its distal segment pre-inflated under varying pressures while maintaining stable force despite pre-inflation differences. **c**, Pull-out force evaluation of the prosthetic hand. The pull-out force is measured by pulling out a power-grasped ball (60-mm diameter) out of the hand mechanism in different directions. B0, B45, B90, B135, and B180 denote the force under pull angles of 0°, 45°, 90°, 135°, 180°, respectively. B-Z denotes the force along the direction vertically out to the palm. **d**, Grasping force evaluation. CH and CV denote the force of a power-grasped cylinder (55-mm diameter) pulled out along the horizontal and vertical directions, respectively. UTM, universal testing machine. **e**, Temporal variations in horizontal and vertical grasping forces.

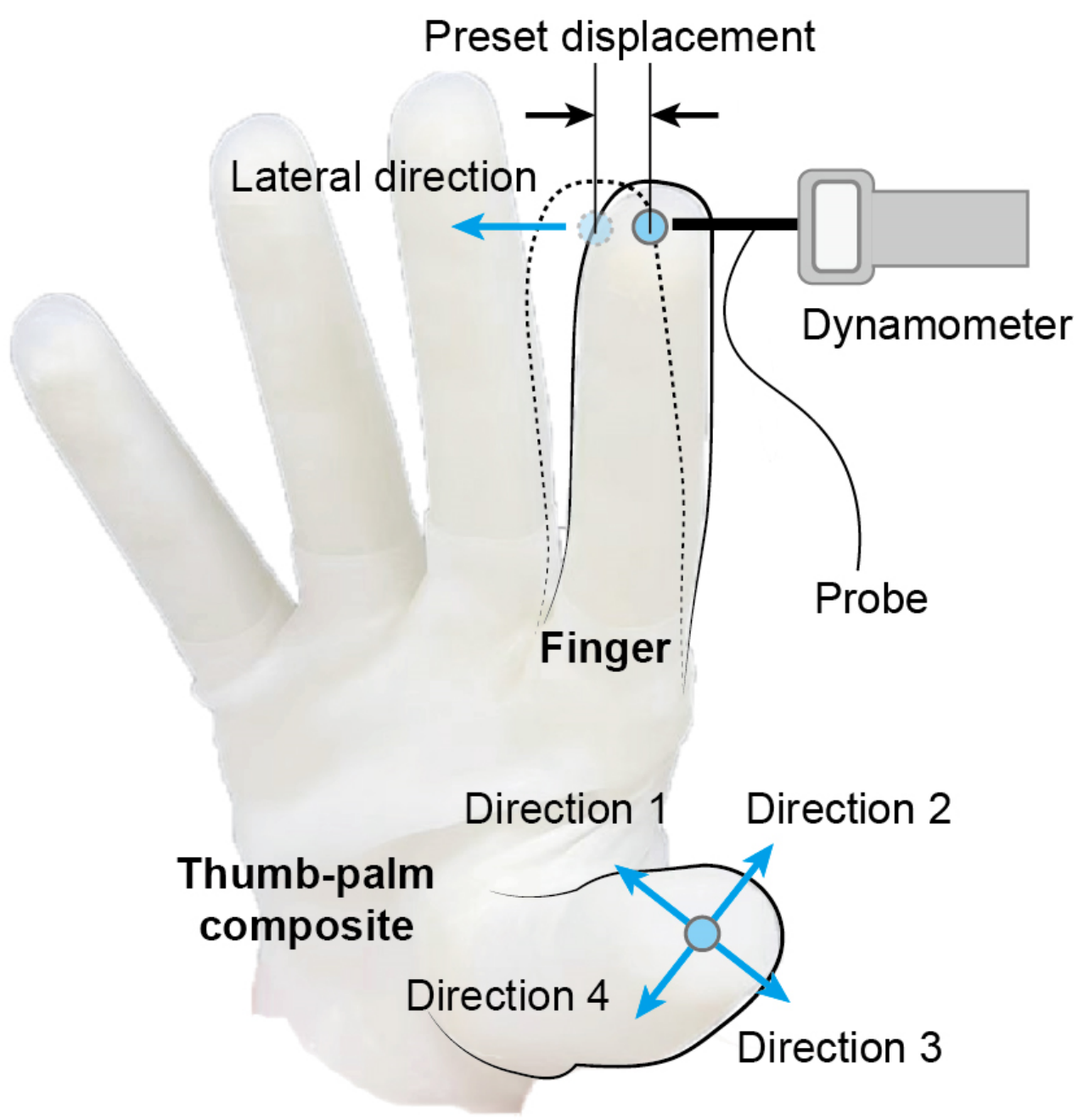

Supplementary Figure 11. Stiffness measurement setup for the finger and thumb-palm composite. Finger (lateral) and thumb-palm composite (four directions) stiffnesses are evaluated by applying a preset displacement (Δd = 10 mm) and measuring the force (f) from a dynamometer with a probe. Stiffness is given by $k = f / \Delta d$.

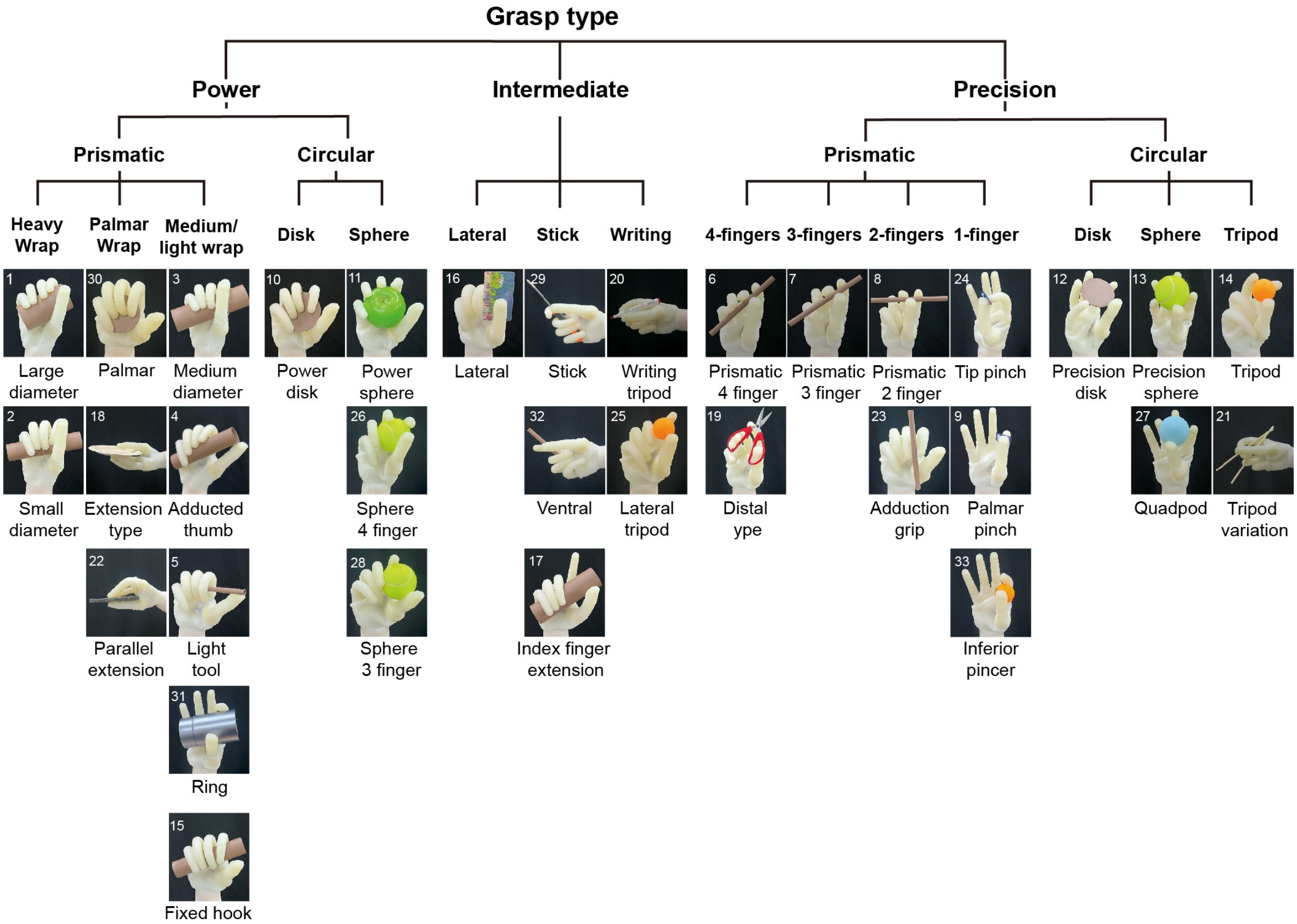

Supplementary Figure 12. Grasping dexterity. The high-dexterity soft neuroprosthetic hand achieves 33 grasp types defined in the GRASP Taxonomy[7].

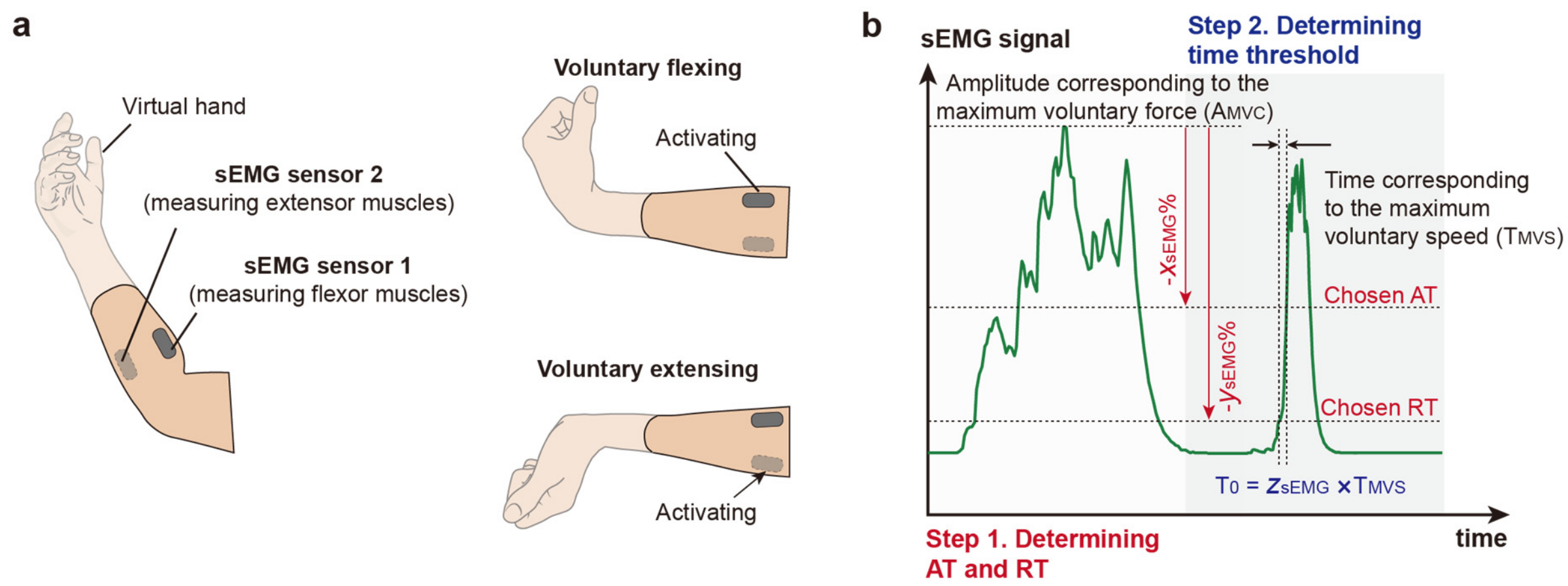

Supplementary Figure 13. Myoelectric control algorithm and parameter determination method. a, Experimental setups and layout of the two sEMG sensors interfacing with the amputee's residual limb. Sensors 1 and sensor 2 measure the flexor and extensor muscles, respectively. The amputee subject is instructed to perform maximal voluntary contractions (MVCs) of both the flexor and extensor muscles by imagining the motion of a virtual hand. **b**, Calibration of subject-specific sEMG threshold parameters. During calibration, the amplitudes (denoted as A_{MVC}) are firstly recorded. To ensure robust and comfortable muscle activation, specific percentages of the MVCs are then set as the action threshold (AT, denoted as $(1\text{-}x_{sEMG}\%) \times A_{MVC}$) and the rest threshold (RT, denoted as $(1\text{-}y_{sEMG}\%) \times A_{MVC}$). The factors x_{sEMG} and y_{sEMG} are identified based on the amputee's subjective feedback to avoid unexpected misjudgment during calibration. Once AT and RT are determined, the subject is instructed to contract the muscles at maximum voluntary speed (MVS), and the time interval (denoted as T_{MVS}) between the instances when the signal amplitude exceeds RT and reaches AT is recorded. The time threshold (T_0) is calculated as $T_0 = z_{sEMG} \times T_{MVS}$. The factor z_{sEMG} is identified based on the amputee's subjective feedback during calibration and remains fixed for subsequent experiments, ensuring a consistent and reliable distinction between the fast active (FA) and slow active (SA) states. Notably, the time threshold T_0 serves as the criterion to distinguish FA and SA contractions.

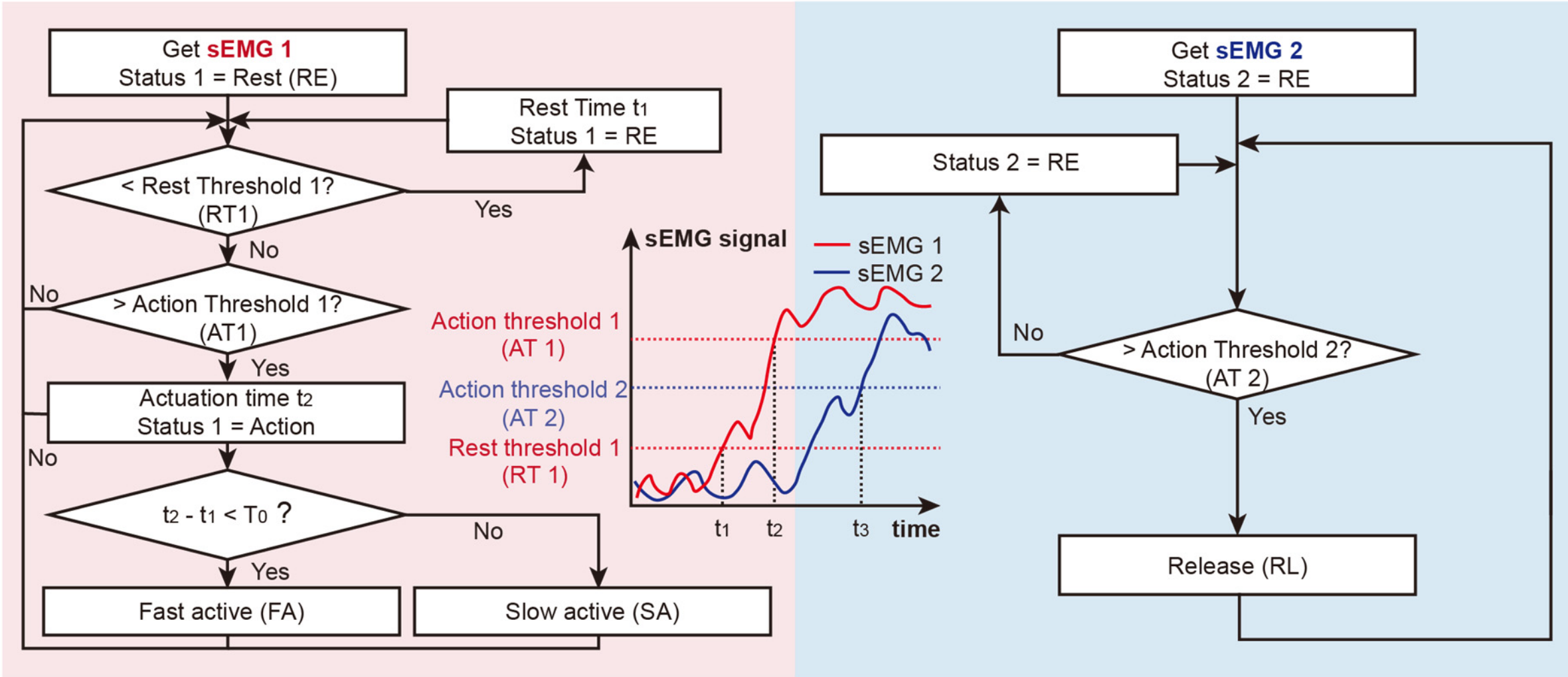

Supplementary Figure 14. Two-channel analysis algorithm. The signal of the flexor muscles (sEMG 1) is mainly used to generate commands for hand motions. The two thresholds (RT 2 and AT 2) of the extensor muscles (sEMG 2) are merged into a single consolidated threshold (AT 2) to ensure robust command Release (RL). t_1, when sEMG 1 surpasses RT 1, t_2: when sEMG 1 reaches AT 1, t_3: when sEMG 2 surpasses AT 2.

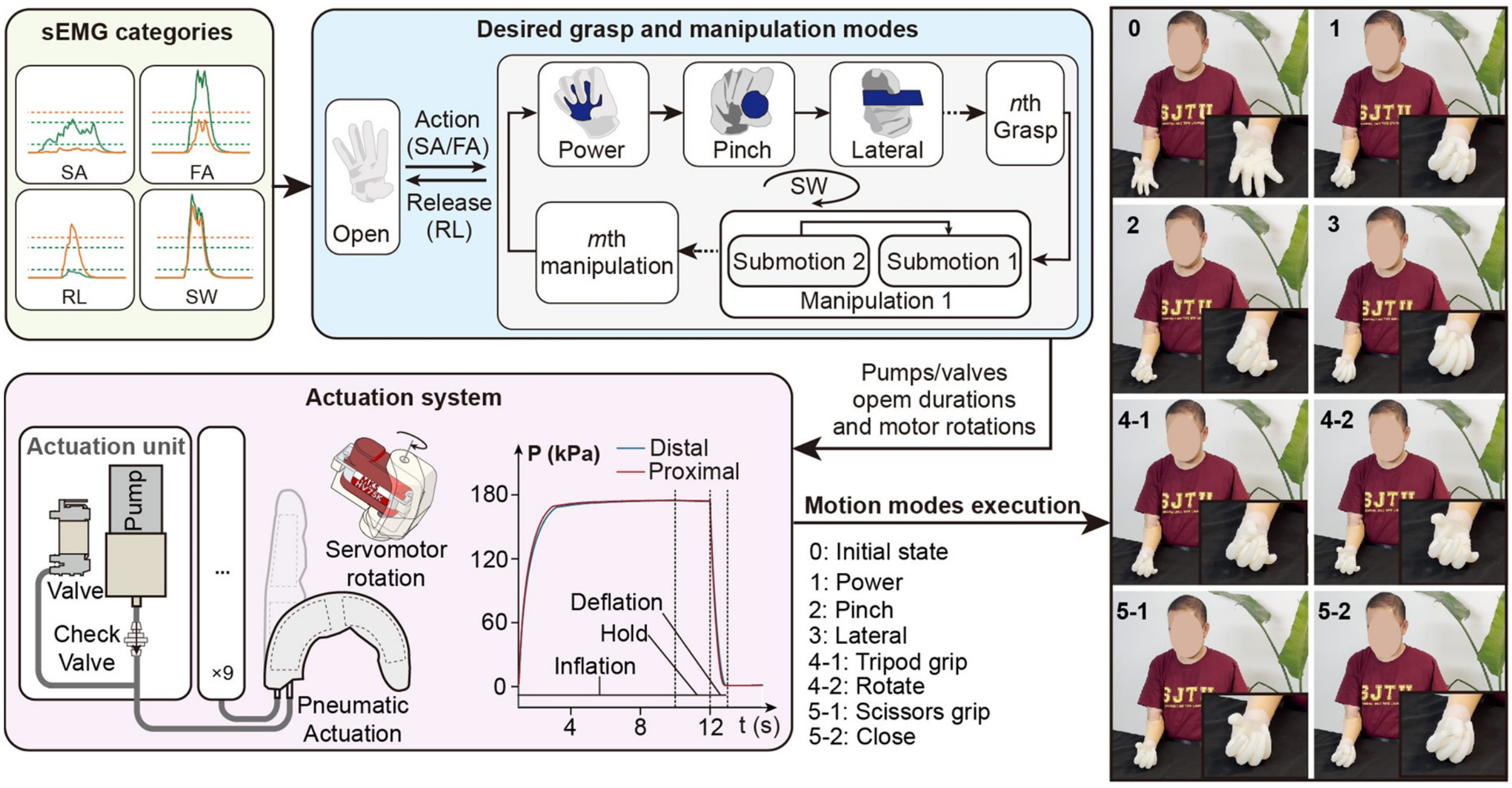

Supplementary Figure 15. Control workflow and demonstration with an amputee subject. This workflow enables the sequential transition between *m*+*n* functional modes (i.e., *n* grasps and *m* manipulations). As a representative example, a functional set comprising 3 grasp modes and 2 manipulation modes is illustrated. The number of modes can be flexibly expanded based on the amputee's individual requirements. Once a functional mode is selected by the amputee subject via sEMG signals, the corresponding control signals of pumps, valves, and the servomotor are generated to execute the functional mode. The effectiveness of this control framework is demonstrated by an amputee subject successfully performing and transitioning between functional modes: grasps (1-Power, 2-Pinch, 3-Lateral) and manipulations (4-scissors use, 5-bulb installation), starting from the initial state (0-hand open).

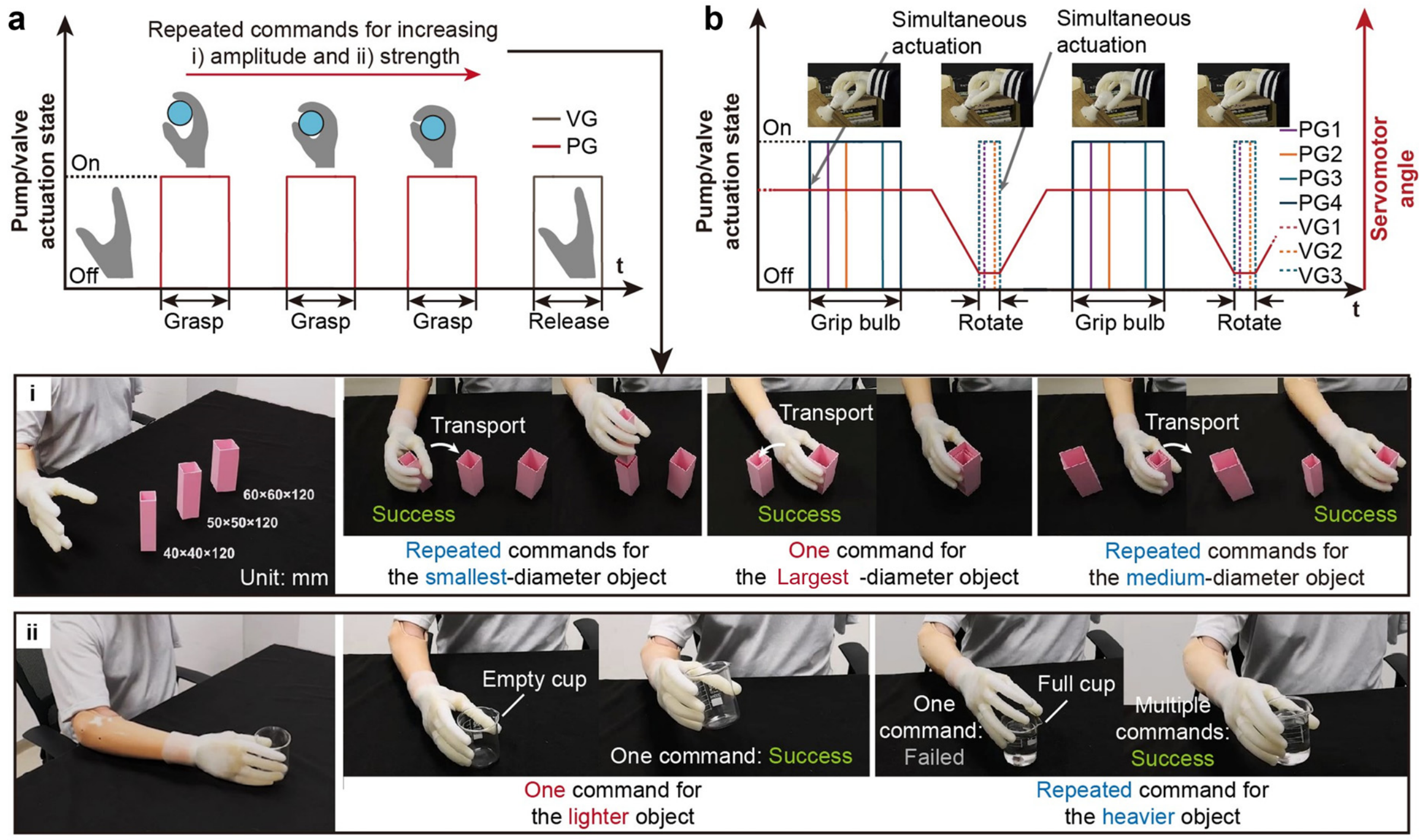

Supplementary Figure 16. Control framework for grasping and manipulation functions. **a**, Grasping amplitude/strength adjustment via repeated commands. These capabilities are demonstrated by an amputee subject performing two tasks in Power grasp mode: 1) grasping cuboid columns with varying cross-sectional diameters, and 2) lifting cups with different weights. **b**, Generation of submotion sequences for manipulation. Actuation signals for the pump groups, valve groups, and servomotor can be programmed to achieve these submotion sequences. PG, pump group. VG, valve group.

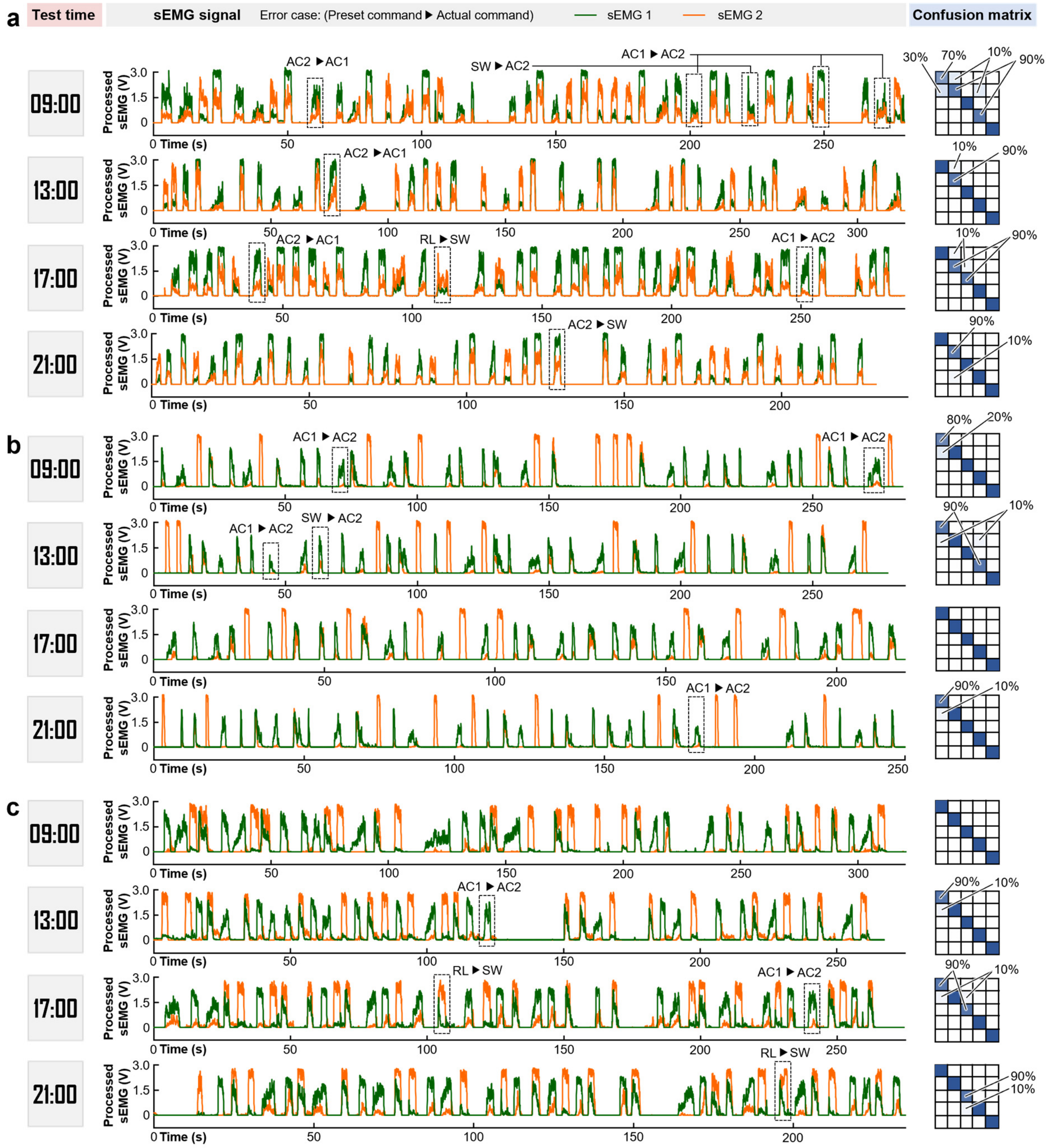

Supplementary Figure 17. 12-hour control performance analysis on amputee subjects. a-c, Results from amputee subjects 2-4. The performance is tested at 3-hour intervals with $n = 50$ preset control commands (detailed in Supplementary Table 8). Subject-specific sEMG signal thresholds are provided in Supplementary Table 9.

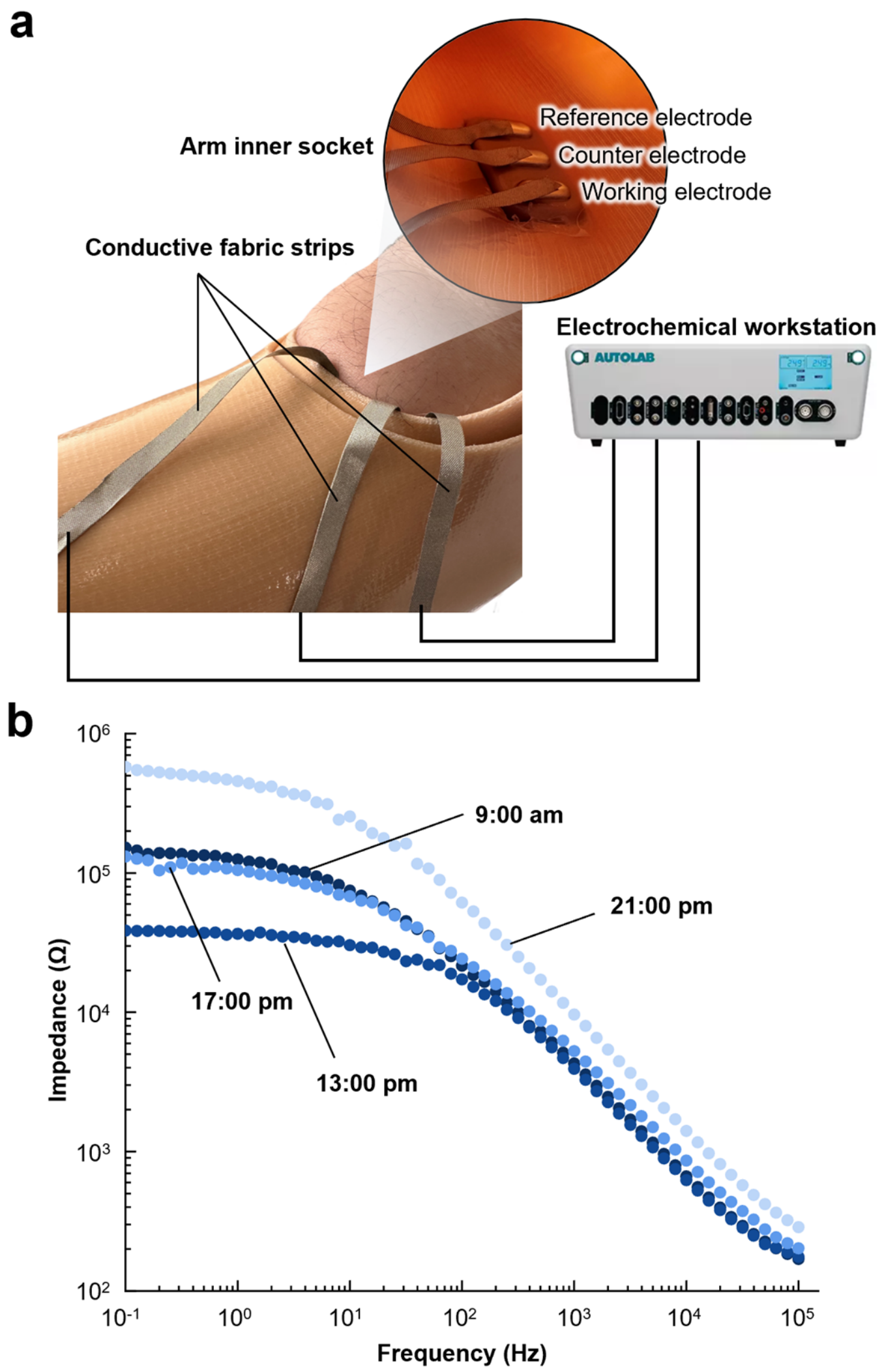

Supplementary Figure 18. Skin impedance evaluations with the sEMG sensor. a, Experimental setups on the amputee subject 1. **b**, Skin impedances as functions of the frequency. The impedance measurements are performed at 4-hour intervals within 12 hours.

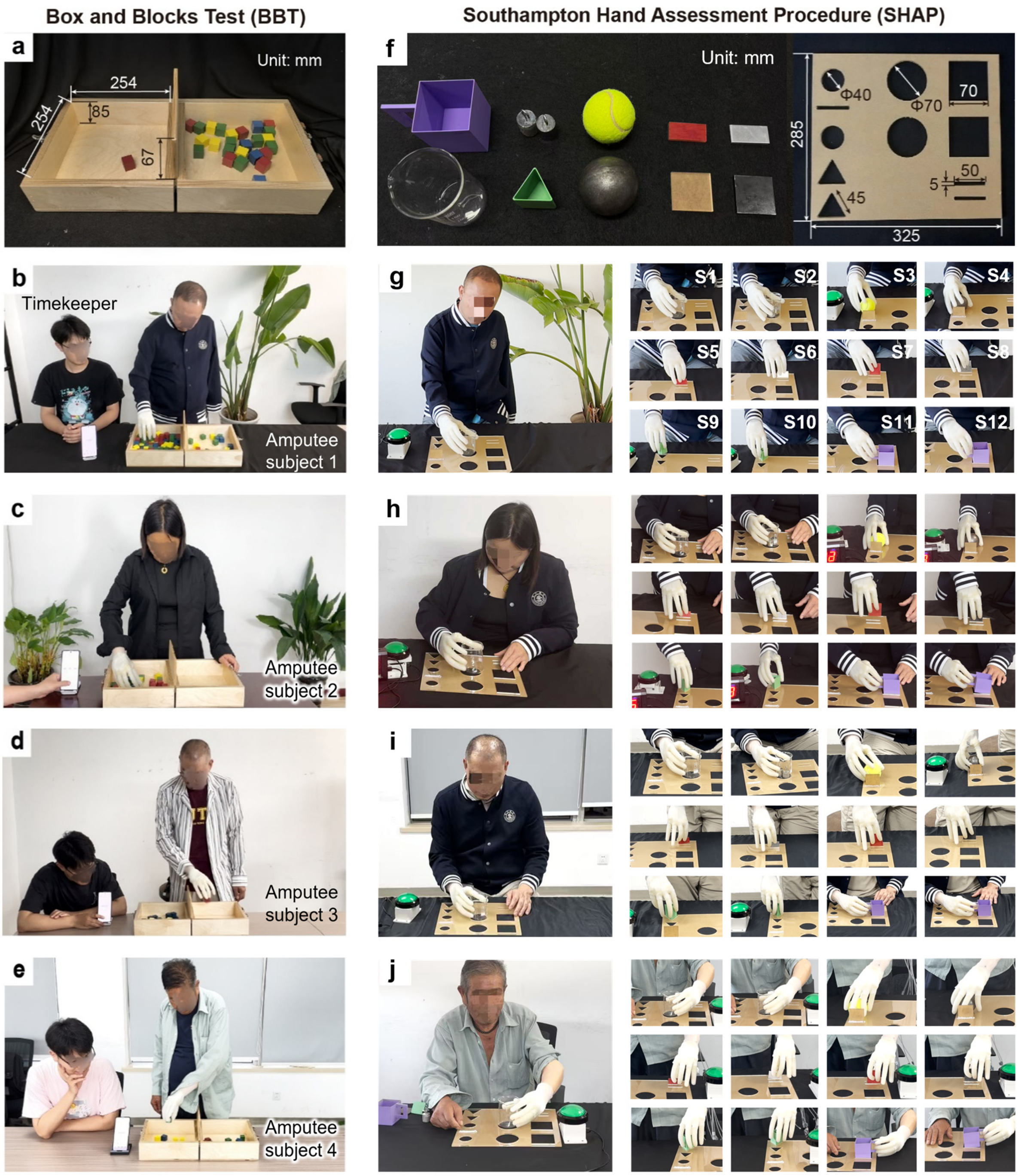

Supplementary Figure 19. Experiment setups and processes of Box and Blocks Test (BBT) and Southampton Hand Assessment Procedure (SHAP). a, BBT setups. **b-e**, Demonstration of four amputee subjects participating in the BBT. The number of blocks grasped and transported within one minute is recorded (n = 3 trials). **f**, SHAP setups. **g-j**, Demonstration of four amputee subjects participating in the SHAP. All four subjects successfully complete the full set of tasks (S1-S12). For each task, the completion time was self-recorded by the subject using a push-button timer (n = 3 trials per task).

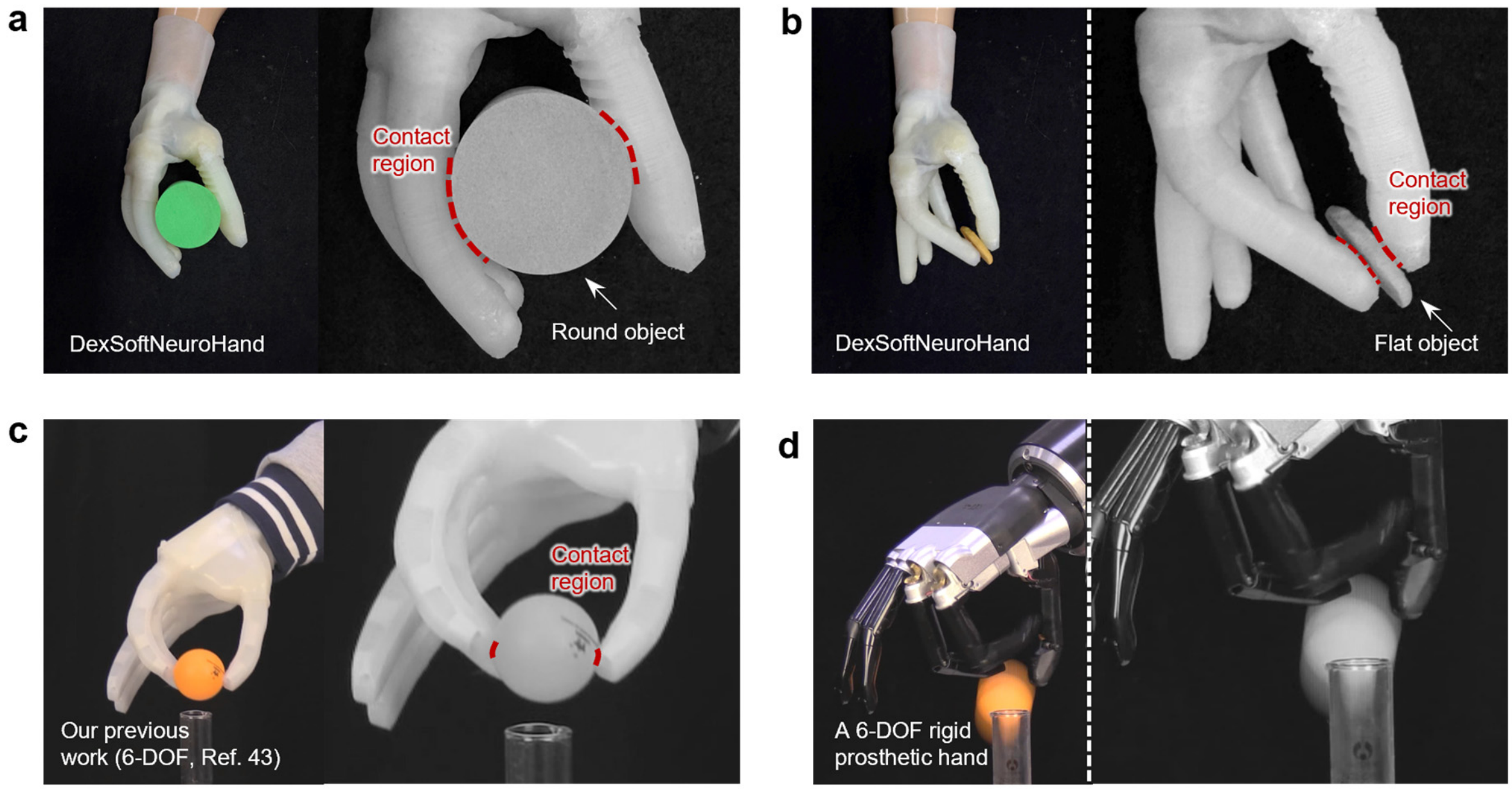

Supplementary Figure 20. Grasping advantages. a-b, The neuroprosthetic hand enables adaptive contact with sufficient regions during grasping through high-DOF coordination, as seen in tasks like power grasping a round object or pinching a flat one. **c**, The 6-DOF soft neuroprosthetic hand from our previous work, typically with only one active flexion DOF per finger, performs pinches with limited contact areas that can lead to instability[42]. **d**, During pinching, a 6-DOF rigid prosthetic hand exhibits limited contact areas and a lack of compliance.

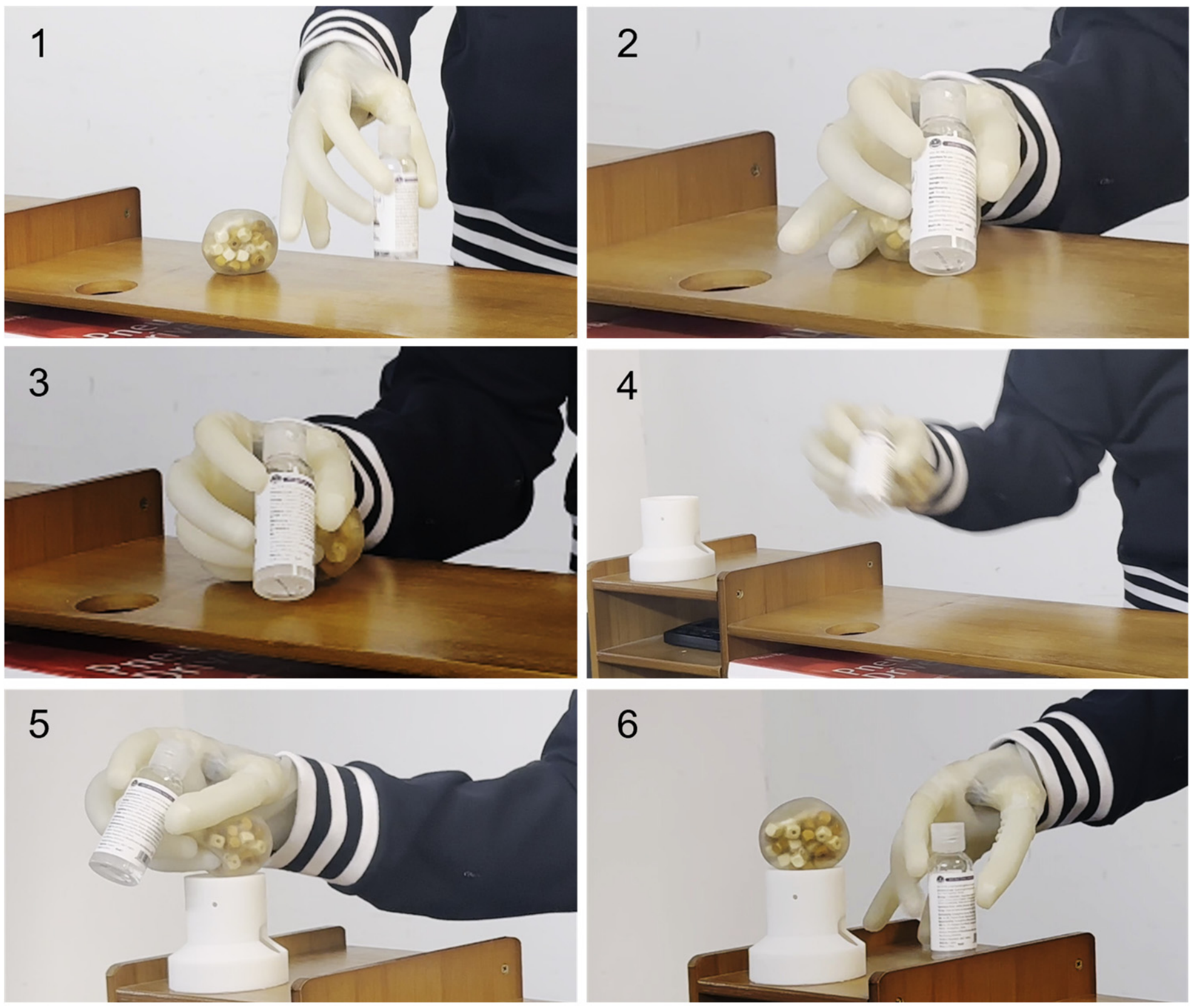

Supplementary Figure 21. Pick and place of two objects. The neuroprosthetic hand enables the amputee to pick and retain one object within the hand while picking a second one, followed by sequentially placing both, unlike the conventional prostheses that need releasing an object before picking up a new one (see Supplementary Video 14 for details).

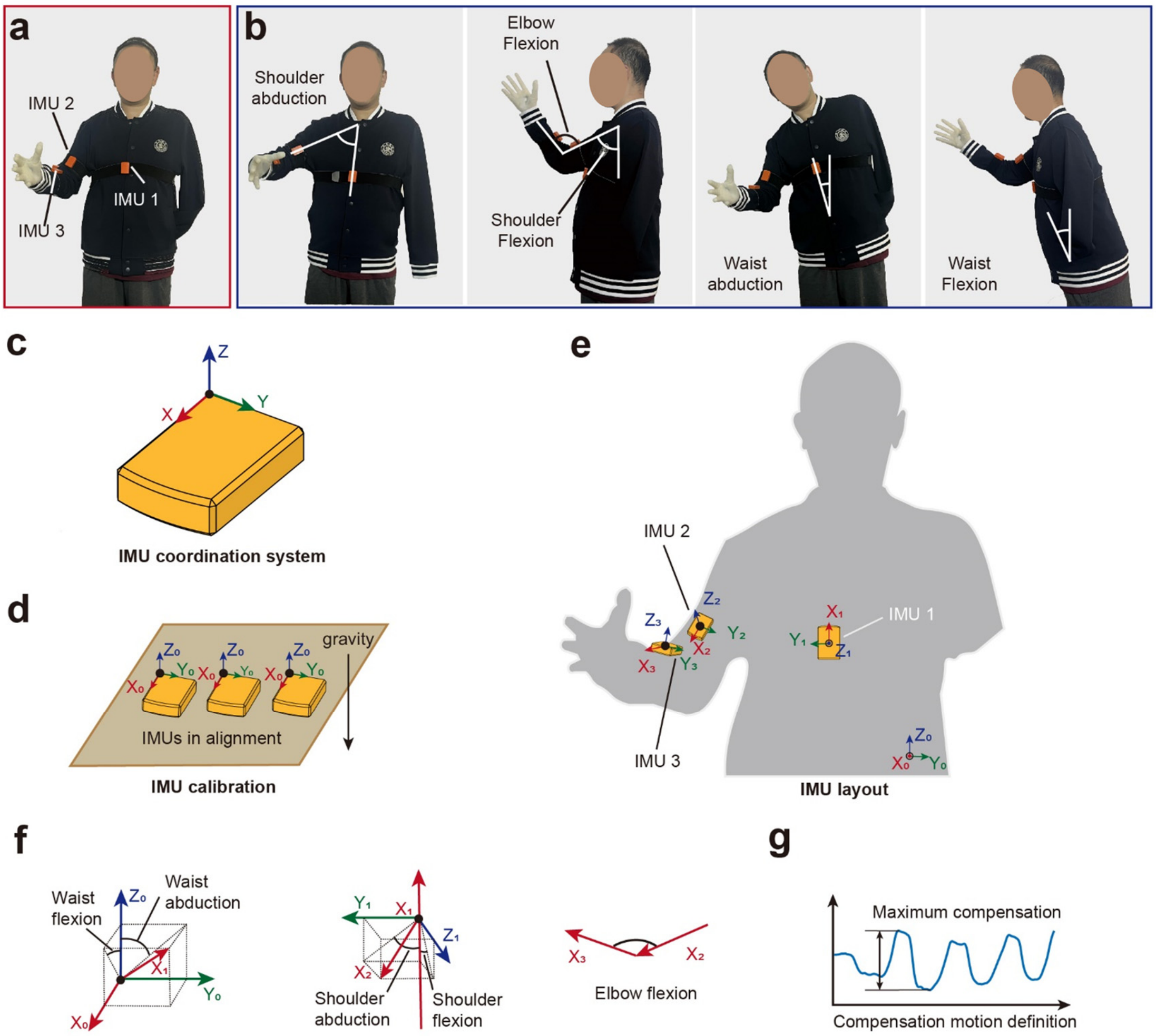

Supplementary Figure 22. Measurement and calculation of the body postural compensation angles. a, The IMU sensor arrangement on the subject to measure the body postural compensation angle. **b**, The definition of the main body compensation angles. **c**, The coordination system of the IMU sensor. **d**, The calibration process of the IMU sensors before the experiment. All the IMU sensors are put in alignment and calibrated to share the same coordinate system layout with the Z_0 axis opposite the gravity direction. **e**, The IMU layout on the amputee subject and the corresponding coordinate systems. The IMU 1 is put on the chest of the subject to measure the waist compensation angles with its X_1 axis parallel to the main body's central axis. The IMU 2 and IMU 3 are put on the upper limb and forearm, respectively, with their X-axes parallel to the corresponding limbs to measure the compensation angles of the shoulder and elbow. **f**, The calculation method of the five body compensation angles. **g**, The maximum compensation angle is defined as the difference between the maximum and minimum joint angle of the joint angle curve during the manipulation task.

Supplementary Table 1

Comparison of our neuroprosthetic hand with existing prosthetic hands in key design characteristics. SMA, Shape-memory alloy; sEMG, surface electromyography; FSM, finite state machine; PR, pattern recognition; NP, not provided; *, the glove weight is not included; #, these systems are developed as commercial products; consequently, their sEMG sensor configurations and algorithms are proprietary and subject to change across firmware updates.

	**Actuation method**	**Active DOFs**	**Self-adapting mechanisms**	**Control method**	**Weight (g)**
Michelangelo (Ref. 2)	Motor +linkage	2	Linkages with local compliant elements	sEMG#	510*
Vincent (Ref. 15)	Motor +linkage	6	Linkages with local compliant elements	sEMG#	485*
i-Limb (Ref. 2)	Motor +linkage	6	Linkages with local compliant elements	sEMG#	628*
Bebionic (Ref. 15)	Motor +linkage	5	Linkages with local compliant elements	sEMG#	588*
Hannes (Ref. 16)	Motor +tendon	1	Rigid skeletons with synergistic elements	sEMG	480*
Pisa/IIT hand (Ref. 18)	Motor +tendon	1	Dislocatable joint +rigid skeletons	NP	NP
Pisa/IIT hand 2 (Ref. 19)	Motor +tendon	2	Dislocatable joint +rigid skeletons	sEMG	~500*
X-Limb (Ref. 38)	Motor +tendon	5	flexure joint +semi-rigid skeletons	NP	253
X hand (Ref. 14)	Motor +tendon	2	NP	NP	NP
DEKA (Ref. 13)	Motor +linkage	4	NP	sEMG	1400
Smart hand (Ref. 11)	Motor +tendon	4	NP	sEMG	530*
SSSA-Myhand (Ref. 12)	Motor +linkage	3	NP	sEMG	478*
SoftHand Pro-2 (Ref. 23)	Motor +tendon	2	Dislocatable joint +rigid skeletons	sEMG	NP
HANDSON (Ref. 32)	Motor +linkage	3	Linkages with local compliant elements	sEMG	NP
19-DOF hand (Ref. 33)	SMA +tendon	19	NP	Voice	220*
Hybrid hand (Ref. 46)	Pneumatic	14	Soft joints +rigid skeletons	sEMG	NP
Our prior work (Ref. 43)	Pneumatic	6	Soft continuous body+rigid skeletons	sEMG	Hand mechanism: 292<br>Total: 736
**This work**	**Pneumatic +motor**	**11**	**Soft continuous body +rigid revolute joint and skeletons**	**sEMG**	**Hand mechanism: 242<br>Total: 703**

Supplementary Table 2

Comparison of our neuroprosthetic hand with existing prosthetic hands in mechanical performances. NP, not provided; F-E, flexion-extension; A-A, abduction-adduction.

	**Active finger kinematics**	**Compliance directions**	**Finger force (N)**	**Hand payload (N)**	**Finger speed (°/s)**
Michelangelo (Ref. 2)	Coupled motion of all fingers	F-E	NP	78.14	86.9
Vincent (Ref. 15)	Coupled MCP/PIP flexion	F-E	3~8.44	NP	87.9~103.3
i-Limb (Ref. 2)	Coupled MCP/PIP flexion	F-E	3.09~11.18	71.44	60.5~110.6
Bebionic (Ref. 15)	Coupled MCP/PIP flexion	F-E	12.25~16.11	77.37	36.6~96.4
Hannes (Ref. 16)	Coupled multi-joint motions	F-E, A-A	NP	51~141	150
Pisa/IIT hand (Ref. 18)	Coupled multi-joint motion	Omni-directional	NP	28	NP
Pisa/IIT hand 2 (Ref. 19)	Coupled multi-joint motion	Omni-directional	NP	NP	NP
X-Limb (Ref. 38)	Coupled multi-joint flexion	Mainly F-E	NP	21.5	NP
X hand (Ref. 14)	Coupled four fingers' motion	Differential mechanism	NP	8.82	NP
DEKA hand (Ref. 13)	NP	NP	NP	NP	NP
Smart hand (Ref. 11)	Coupled multi-joint motion	Mainly F-E	NP	98	NP
SSSA-Myhand (Ref. 12)	Coupled multi-joint motion	NP	11.7~31.4	NP	160~170
SoftHand Pro-2 (Ref. 23)	Decoupled thumb abduction + coupled all finger flexion	F-E	NP	NP	NP
HANDSON (Ref. 32)	Two-finger gripper with coupled flexion	NP	NP	NP	NP
19-DOF hand (Ref. 33)	Decoupled MCP and PIP flexion + MCP abduction	Omni-directional	NP	24.5	63
Hybrid hand (Ref. 46)	Decoupled MCP, PIP and DIP flexion	NP	1.8	>16	NP
Our prior work (Ref. 43)	Coupled MCP, PIP and DIP flexion	Omni-directional	1.9	>20	105
**This work**	**Decoupled MCP and (PIP+DIP) flexion**	**Omni-directional**	**Thumb: 3.5 Finger: 2.6**	**>25**	**110**

Supplementary Table 3

Comparison of our neuroprosthetic hand with existing prosthetic hands in key functionalities and amputee validations. NP, not provided.

	Grasping	Manipulation	Amputee validation
Michelangelo (Ref. 2)	Lateral, palmar	No	Post-commercially validated
Vincent (Ref. 15)	NP	No	Post-commercially validated
i-Limb (Ref. 2)	Lateral, palmar, power	No	Post-commercially validated
Bebionic hand (Ref. 15)	Lateral, palmar, power	No	Post-commercially validated
Hannes (Ref. 16)	Lateral, precision, power	No	3 male amputees, 29~62 years old
Pisa/IIT hand (Ref. 18)	Synergistic grasps	No	Able-bodied individuals fitted with a bypass prosthesis
Pisa/IIT hand 2 (Ref. 19)	Synergistic grasps	Non-amputee demonstration: object rotation, jar opening, coffee pouring	Able-bodied individuals fitted with a bypass prosthesis
X-Limb (Ref. 38)	Adaptive grasps: pinch, tripod, power	No	Able-bodied individuals fitted with a bypass prosthesis
X hand (Ref. 14)	29 human-like postures	Non-amputee demonstration: ball/tweezer manipulation, cap screwing	No
DEKA hand (Ref. 13)	Pinch, tripod, power, lateral, precision	No	21 amputees, 26~65 years old
Smart hand (Ref. 11)	Pinch, power, lateral, precision, tool, chuck	No	NP
SSSA-Myhand (Ref. 12)	Power, lateral, precision	No	NP
SoftHand Pro-2 (Ref. 23)	Power, pinch, tripod	Simple in-hand manipulation with tethered devices	3 female amputees, 37.3±3.5 years old
HANDSON (Ref. 32)	power, pinch, precision	Bulb installation	1 female amputee, 50 years old
19-DOF hand (Ref. 33)	33 human-like postures	Non-amputee demonstration: syringe and scissor use	1 female amputee
Hybrid hand (Ref. 46)	Spherical, pinch, power, tripod, cylindrical (demonstration with tethered devices)	No	Robotic arm or able-bodied individuals fitted with a bypass prosthesis
Our prior work (Ref. 43)	Power, pinch, lateral, precision disk	No	2 male amputees
**This work**	**33 human-like adaptive postures, safe and compliant interaction**	**Continuous scissors use, pick and place two objects, Bulb installation**	**4 amputees, 38~70 years old, 3 males, 1 female**

Supplementary Table 4

Detailed information of our neuroprosthetic hand's main components.

Component	Dimension (mm×mm×mm)	Weight (g)	Materials and parameters of the component	Price (USD)
**Hand mechanism (total weight of 242 g)**				
Thumb	100×24×20	20	Elastomeric chamber: Dragonskin 20; Skin: Ecoflex 0030;	3
Index, middle, ring, and little fingers	110×24×20 (per piece)	78 (4 pieces)		12
Thumb-palm connection linkage	75×50×20	36.5	3D printed with resin (8200, Wenext)	3
Palm with skin	94×78×132	100	Palm skeleton: 3D printed with resin (8200, Wenext); Skin: fabricated from Ecoflex 0035	22
Servomotor	23×9×16.7	7.5	Speed: 0.09s; Torque: 3.3 kg-cm	81.25
**Pneumatic control systems in a waist bag (total weight of 461 g)**				
sEMG recognition and collection unit	45×18×7.2	10 (2 pieces)	Arduino (nano) 5-12 V, 12-channel input/output pins	22
Pneumatic control unit	78×50×7.8	15	Arduino (mega)	20
Data storage unit	24×30×1	2	Voltage: 5 V	1
Timing module	38×22×14	8	Zave (DS3231) Voltage: 3.3-5.5V	0.9
PCB circuit board	80×50×1.1	17	Customized	25
Pump	67×27×27	302 (10 pieces)	Maximum pressure: 185 kPa; Flow rate: 3.5 LPM; Voltage: 6 V	50
Valve	23×12×8	38 (10 pieces)	Parker (X-valve), 2-way normal closed, directional, 12 V	350
Voltage regulator	18×10×2.6	2	Input voltage range: 4-36 V	~6
Battery	57×29×22	67	Voltage: 11.1 V	~10
**Customized arm socket**				
Socket	274×95×90	75~105	Customized	~30
sEMG sensors	35×22×10	10 (2 pieces)	Danyang Inc.	~100
Passive wrist	45×45×19	25	Customized	~9

Supplementary Table 5

Stiffness characteristics of hand components. Soft fingers with different chamber materials under 200-kPa actuation pressure show varied stiffnesses. The material Dragonskin 20 is selected as it provides higher finger stiffness for improved operational stability while achieving sufficient bending angles within portable actuation constraints.

**Material of the finger inner chamber**	**Measured finger stiffness**	**Finger bending angle**
Ecoflex 0030	8.2 ± 0.8 N/m	356.4°
Dragonskin 10	33.4 ± 1.7 N/m	197.8°
Dragonskin 20	61.0 ± 2.2 N/m	183.2°
Dragonskin 30	89.3 ± 3.1 N/m	75.6°
**Measured stiffness**	**Our previous work***	**This work**
Finger	24.6 ± 3.8 N/m	61.0 ± 2.2 N/m
Thumb-palm composite (direction 1)	15.0 ± 0.8 N/m	169.0 ± 7.3 N/m
Thumb-palm composite (direction 2)	12.7 ± 1.2 N/m	168.3 ± 6.6 N/m
Thumb-palm composite (direction 3)	16.7 ± 1.2 N/m	168.0 ± 5.4 N/m
Thumb-palm composite (direction 4)	15.3 ± 1.7 N/m	163.5 ± 10.9 N/m

*Gu, G. et al. A soft neuroprosthetic hand providing simultaneous myoelectric control and tactile feedback. *Nat. Biomed. Eng.* 7, 589-598 (2023).

Supplementary Table 6

Control signal of grasp types. DOF 1, motor angle; DOF 2 to DOF 11, pump open time.

	DOF 1 (°)	DOF 2 (ms)	DOF 3 (ms)	DOF 4 (ms)	DOF 5 (ms)	DOF 6 (ms)	DOF 7 (ms)	DOF 8 (ms)	DOF 9 (ms)	DOF 10 (ms)	DOF 11 (ms)
1.Large diameter	45	1000	1000	1000	1000	1000	1000	1000	1000	1000	1000
2.Small diameter	45	1000	1000	1000	1000	1000	1000	1000	1000	1000	1000
3.Medium diameter	45	1000	1000	1000	1000	1000	1000	1000	1000	1000	1000
4.Adducted thumb	30	0	0	1000	1000	1000	1000	1000	1000	1000	1000
5.Light tool	30	0	0	1000	1000	1000	1000	1000	1000	1000	1000
6.Prismatic 4 fingers	45	500	500	500	250	500	250	500	250	500	250
7.Prismatic 3 fingers	45	500	500	500	500	500	500	1000	1000	1000	1000
8.Prismatic 2 fingers	45	500	500	500	500	1000	1000	1000	1000	1000	1000
9.Palmar pinch	45	600	600	600	600	0	0	0	0	0	0
10.Power disk	30	1000	1000	1000	1000	1000	1000	1000	1000	1000	1000
11.Power sphere	30	1000	1000	1000	1000	1000	1000	1000	1000	1000	1000
12.Precision disk	45	500	500	500	500	500	500	500	500	500	500
13.Precision sphere	45	500	500	500	500	500	500	500	500	500	500
14.Tripod	45	500	500	500	500	500	500	1000	1000	1000	1000
15.Fixed hook	30	0	0	1000	1000	1000	1000	1000	1000	1000	1000
16.Lateral	30	1000	1000	1000	1000	1000	1000	1000	1000	1000	1000
17.Index finger extension	30	1000	0	0	0	1000	1000	1000	1000	1000	1000
18.Extension type	30	1000	0	0	0	0	0	0	0	0	0
19.Distal type	45	500	500	500	250	600	300	700	350	800	800
20.Writing tripod	45	500	500	500	250	500	250	0	0	0	0
21.Tripod variation	45	500	500	500	500	500	500	1000	1000	1000	1000
22.Parallel extension	45	0	0	750	0	750	0	750	0	750	0
23.Adduction grip	30	0	0	1000	1000	1000	1000	1000	1000	1000	1000
24.Tip pinch	45	600	600	600	600	0	0	0	0	0	0
25.Lateral tripod	45	500	500	500	500	500	500	1000	1000	1000	1000
26.Sphere 4 finger	45	1000	1000	1000	1000	1000	1000	1000	1000	1000	1000
27.Quadpod	45	500	500	500	500	500	500	500	500	0	0
28.Sphere 3 finger	45	1000	1000	1000	1000	1000	1000	1000	1000	1000	1000
29.Stick	45	600	600	1000	1000	1000	1000	1000	1000	1000	1000
30.Palmar Wrap	30	1000	1000	1000	1000	1000	1000	1000	1000	1000	1000
31.Ring	45	1000	1000	1000	1000	0	0	0	0	0	0
32.Ventral	45	600	200	1000	1000	1000	1000	1000	1000	1000	1000
33.Inferior pincer	45	600	600	600	600	0	0	0	0	0	0

Supplementary Table 7
Basic information of the amputee subjects.

Amputee	Age	Gender	Amputation	Prosthetic utilization	Frequent life scenarios
Subject 1	38	Male	Right hand (below elbow disarticulation), Left hand (shoulder disarticulation)	Full-time	Independent living ability (e.g., dressing, feeding and cleaning tasks)
Subject 2	42	Female	Right hand (wrist disarticulation)	Part-time	Household chores, intimate interaction in family caregiving roles, water-resistant functionality
Subject 3	46	Male	Right hand (wrist disarticulation)	No	Operation in urban life (e.g., making coffee, driving and recreational activities)
Subject 4	70	Male	Left hand (wrist disarticulation)	No	Life assistant (e.g., taking medicine)

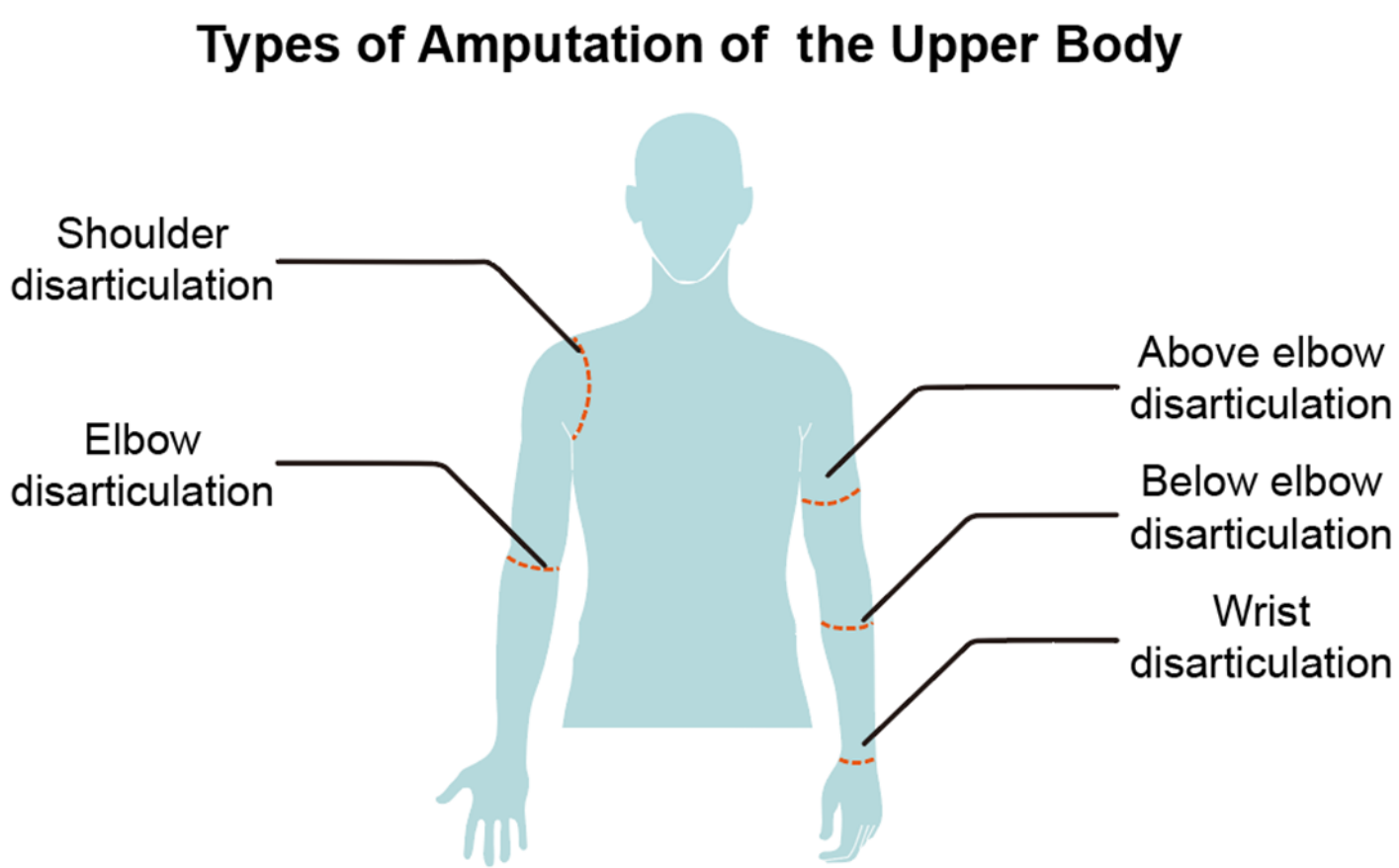

Supplementary Table 8

The preset sEMG commands for 12-hour myoelectric tests, which are conducted at time sessions: 9:00, 13:00, 17:00, and 21:00. At each session, each amputee subject is instructed to perform a total of 50 preset sEMG commands in order, including 10 AC1s, 10 AC2s, 10 RLs, 10 SWs, and10 KEs.

**Test time: 9:00**										
**Command 1-10**	AC2	AC1	RL	SW	KE	AC2	AC1	RL	SW	KE
**Command 11-20**	AC2	KE	AC2	AC1	SW	RL	AC1	SW	RL	KE
**Command 21-30**	AC1	AC2	KE	AC2	SW	AC1	RL	KE	SW	RL
**Command 31-40**	RL	KE	RL	SW	AC1	AC2	AC1	KE	SW	AC2
**Command 41-50**	KE	AC1	SW	AC2	KE	RL	SW	AC2	AC1	RL
**Test time: 13:00**										
**Command 1-10**	RL	KE	RL	AC2	SW	AC1	KE	SW	AC2	AC1
**Command 11-20**	AC1	SW	AC2	AC1	KE	RL	AC2	SW	KE	RL
**Command 21-30**	AC2	RL	KE	AC1	RL	SW	AC2	KE	AC1	SW
**Command 31-40**	SW	AC1	RL	KE	SW	AC2	AC1	RL	KE	AC2
**Command 41-50**	KE	AC2	SW	AC1	RL	KE	AC2	SW	AC1	RL
**Test time: 17:00**										
**Command 1-10**	AC1	KE	AC2	AC1	SW	RL	KE	AC2	RL	SW
**Command 11-20**	SW	AC2	RL	SW	KE	AC1	AC2	RL	KE	AC1
**Command 21-30**	RL	KE	AC1	RL	AC2	SW	KE	AC1	AC2	SW
**Command 31-40**	AC2	SW	KE	AC2	AC1	RL	SW	KE	AC1	RL
**Command 41-50**	AC1	RL	AC2	KE	AC1	SW	RL	AC2	KE	SW
**Test time: 21:00**										
**Command 1-10**	RL	KE	AC2	SW	RL	AC1	KE	AC2	SW	AC1
**Command 11-20**	SW	AC2	AC1	KE	SW	RL	AC2	AC1	KE	RL
**Command 21-30**	KE	AC1	RL	AC2	KE	SW	AC1	RL	AC2	SW
**Command 31-40**	AC2	SW	KE	AC1	AC2	RL	SW	KE	AC1	RL
**Command 41-50**	RL	AC1	SW	KE	RL	AC2	AC1	SW	KE	AC2

Supplementary Table 9

The sEMG signal thresholds for different amputee subjects.

**Amputee subject**	**AT1 (V)**	**RT1 (V)**	**AT2 (V)**	**Time threshold (ms)**
Subject 1	0.98	0.38	1.28	300
Subject 2	1.13	0.16	1.29	300
Subject 3	0.97	0.10	0.81	200
Subject 4	1.61	0.48	0.97	300

AT1, action threshold of the processed sEMG 1. RT1, rest threshold of the processed sEMG 1. AT2, action threshold of the processed sEMG 2.

Supplementary Table 10

Detailed evaluation results of the Box and Blocks Test.

**Amputee subject**	**Block number**			**Average block number**	**Standard deviation**
	1st	2nd	3rd		
Subject 1	27	29	30	28.67	1.25
Subject 2	25	25	29	26.33	1.89
Subject 3	23	23	22	22.67	0.47
Subject 4	17	19	20	18.67	1.25

Supplementary Table 11

A comparison of BBT scores among this work, our previous work, and current neuroprosthetic hands.

<table>
<tr><th></th><th>Ref. 1*</th><th>Ref. 2**</th><th>Ref. 3***</th><th>Our previous work****</th><th>This work</th></tr>
<tr><td>Number of blocks</td><td>14.3</td><td>26</td><td>24</td><td>16.3</td><td>18.7-28.7</td></tr>
<tr><td></td><td colspan="5">*Resnik, L. et al. Reliability and validity of outcome measures for upper limb amputation. J. Prosthet. Orthot. 24, 192-201 (2012).
**Smit, G., et al. The lightweight Delft Cylinder Hand: first multi-articulating hand that meets the basic user requirements. IEEE Trans. Neural Syst. Rehabil. Eng. 23, 431-440 (2014).
***Phillips, S. L., et al. Experiences and outcomes with powered partial hand prostheses: a case series of subjects with multiple limb amputations. J. Prosthet. Orthot. 24, 93-97 (2012).
****Gu, G. et al. A soft neuroprosthetic hand providing simultaneous myoelectric control and tactile feedback. Nat. Biomed. Eng. 7, 589-598 (2023).</td></tr>
</table>

Supplementary Table 12

Detailed information of the objects used in the Southampton Hand Assessment Procedure.

Task	Object name	Weight (g)	Dimension (mm×mm×mm)
S1	Light cylinder cup	90	90×70×70
S2	Heavy cylinder cup	290	90×70×70
S3	Light sphere	56.9	66.7×66.7×66.7
S4	Heavy sphere	242	70×70×70
S5	Small acrylic sheet	6.6	25×50×5
S6	Small metal sheet	46.8	25×50×5
S7	Large acrylic sheet	13	50×50×5
S8	Large metal sheet	88.2	50×50×5
S9	Light triangular prism	9.6	43×43×50
S10	Heavy triangular prism	109.6	43×43×50
S11	Light cup	30.3	65×65×65
S12	Heavy cup	130.2	65×65×65

Supplementary Table 13

Detailed evaluation results of the SHAP.

Amputee	Task	Completion time (s)			Average completion time (s)	Standard deviation (s)
		1st	2nd	3rd		
Subject 1	S1	2.68	2.64	2.79	2.70	0.06
	S2	2.72	2.56	2.42	2.57	0.12
	S3	2.03	2.07	2.11	2.07	0.03
	S4	2.85	2.81	2.50	2.72	0.16
	S5	3.75	3.48	3.48	3.57	0.13
	S6	3.20	3.38	3.46	3.35	0.11
	S7	3.01	2.60	2.75	2.79	0.17
	S8	3.10	3.13	2.96	3.06	0.07
	S9	2.77	2.64	2.74	2.72	0.06
	S10	3.04	3.09	3.24	3.12	0.08
	S11	3.13	3.30	3.25	3.23	0.07
	S12	3.45	3.17	2.97	3.20	0.20
Subject 2	S1	3.38	3.43	3.21	3.34	0.09
	S2	3.92	4.02	3.68	3.87	0.14
	S3	2.08	2.18	2.03	2.10	0.06
	S4	2.84	2.64	2.83	2.77	0.09
	S5	3.43	3.28	3.34	3.56	0.06
	S6	3.56	3.36	3.76	3.14	0.16
	S7	3.02	3.13	3.28	3.73	0.11
	S8	4.02	3.54	3.64	3.12	0.21
	S9	3.16	3.24	2.95	3.12	0.12
	S10	3.73	3.63	4.10	3.82	0.20
	S11	2.92	3.26	2.96	3.05	0.15
	S12	3.01	2.76	2.65	2.81	0.15
Subject 3	S1	2.65	2.65	2.71	2.67	0.03
	S2	2.67	2.78	2.92	2.79	0.10
	S3	2.42	2.32	2.28	2.34	0.06
	S4	3.17	3.53	3.15	3.28	0.17
	S5	3.49	3.56	3.09	3.38	0.21
	S6	3.54	3.47	3.68	3.56	0.09
	S7	3.21	3.26	3.08	3.18	0.08
	S8	3.75	3.84	3.98	3.86	0.09
	S9	3.08	3.13	3.01	3.07	0.05
	S10	3.37	3.47	3.43	3.42	0.04
	S11	3.99	3.98	3.93	3.97	0.03
	S12	4.45	4.21	4.40	4.35	0.10
Subject 4	S1	2.81	2.86	2.71	2.79	0.06
	S2	2.56	2.67	2.76	2.66	0.08
	S3	3.27	3.21	3.11	3.20	0.07
	S4	3.79	3.52	3.47	3.59	0.14
	S5	3.22	3.37	3.14	3.24	0.10
	S6	3.81	3.97	3.57	3.78	0.16
	S7	3.49	3.22	3.18	3.30	0.14
	S8	4.47	4.12	4.47	4.35	0.16
	S9	3.59	3.44	3.72	3.58	0.11
	S10	3.46	3.34	3.55	3.45	0.09
	S11	3.52	3.79	3.56	3.62	0.12
	S12	3.36	3.61	3.59	3.52	0.11

Supplementary Table 14

Comparison of the completion time of SHAP tasks with our neuroprosthetic hand and current neuroprosthetic hands.

SHAP task	Existing prosthetic hand* (s)	Our previous work** (s)	Subject1 (s)	Subject2 (s)	Subject3 (s)	Subject4 (s)
S1: power light	3.75	4	2.68	3.38	2.65	2.81
S2: power heavy	4.12	4.7	2.56	3.92	2.78	2.67
S3: spherical light	4.31	3.6	2.07	2.08	2.32	3.21
S4: spherical heavy	4.30	4.3	2.81	2.83	3.17	3.52
S5: tip light	4.63	4.6	3.48	3.34	3.49	3.22
S6: tip heavy	4.58	5.3	3.38	3.56	3.54	3.81
S7: extension light	4.75	5.3	2.75	3.13	3.21	3.22
S8: extension heavy	4.38	6.9	3.1	3.64	3.84	4.47
S9: tripod light	4.94	5.1	2.74	3.16	3.08	3.59
S10: tripod heavy	4.25	Failed	3.09	3.73	3.43	3.46
S11: lateral light	5.10	Failed	3.25	2.96	3.98	3.56
S12: lateral heavy	4.97	Failed	3.17	2.76	4.4	3.59

* Burgerhof, J. G. et al. The Southampton hand assessment procedure revisited: A transparent linear scoring system, applied to data of experienced prosthetic users. *J. Hand Ther.* 30, 49-57 (2017).

** Gu, G. et al. A soft neuroprosthetic hand providing simultaneous myoelectric control and tactile feedback. *Nat. Biomed. Eng.* 7, 589-598 (2023).

Supplementary Table 15

Main objects involved in the application experiments of activities in daily living.

Objects	Object Type	Weight (g)	Dimension (mm×mm×mm)	Texture material
Card	Flat	4.1	48×60×2	Plastic
Block	Convex	8	25×25×25	Wood
Water Can	Convex	371.3	164×98×98	Plastic
Tissue	Deformable	1	174×130×0.2	Paper
Pen	Convex	13.9	145×15×15	Plastic
Cucumber	Convex	232	322×28×28	Organic
Coffee pack	Deformable	9.5	42×93×5	Plastic
Thermal mug	Convex	613	221×75×75	Metal
Flower bunch	Deformable	114	310×40×40	Organic
Banana	Convex	163	202×36×36	Organic
Tofu	Deformable	12	20×20×20	Organic
Nail	Convex	2.2	38×2.9×2.9	Metal
Chinese chess pieces	Convex	51	20×57×57	Wood
Chess pieces	Non-convex	46	75×30×30	Wood
Pill bottle	Convex	25	40×24×24	Plastic
String	Deformable	0.5	0.3×0.3×100	Polyethylene
Bag with 2.5-kg weights	Deformable	2503	Not applicable	Plastic
Doll	Convex	100	51×105×128	Fabric
Sea snail	Non-convex	20	16×18×32	Organic
Baseball cap	Deformable	83	202 × 180 × 120	Cotton/Fabric
Headphone stand	Non-convex	312	154 × 150 × 223	Wood
Wireless mouse	Convex	95	111 × 60 × 34	Plastic
Phone stand	Non-convex	52	110 × 72 × 21	Plastic
Folded umbrella	Convex	245	283 × 51 × 50	Fabric/Metal
Game controller	Non-convex	284	155 × 104 × 63	Plastic
Data cable	Deformable	150	~152 × 100 × 42	Rubber/Plastic
Foam ball	Deformable	21	61 × 60 × 61	Foam
Over-ear headphone	Non-convex	255	200 × 182 × 92	Plastic/Leatherette
Green pepper	Convex	124	151 × 50 × 51	Organic
Tube (signagel)	Deformable	280	204 × 81 × 40	Plastic
Glass goblet	Non-convex	180	81 × 80 × 171	Glass
Stapler	Non-convex	198	131 × 40 × 55	Plastic/Metal
Pinecone	Non-convex	40	82 × 61 × 60	Wood/Organic
Phone case	Flat	42	161 × 80 × 12	Plastic/Silicone
Drink cup	Convex	510	96 × 95 × 174	Plastic
Clownfish toy	Deformable	62	120 × 71 × 52	Plastic
Rubber doll	Deformable	50	81 × 73 × 80	Rubber/Vinyl
Grapes	Deformable	300	181 × 100 × 62	Organic
Scalp massager	Non-convex	78	91 × 90 × 80	Plastic/Silicone

Types of Objects *

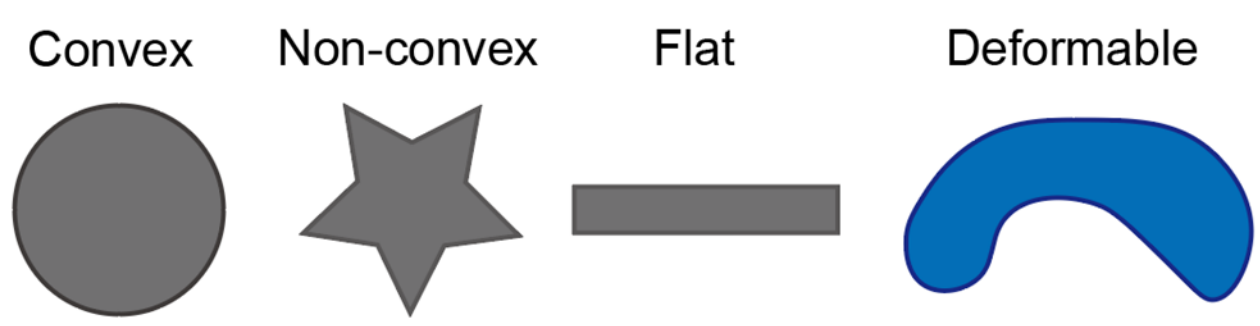

* Shintake, J. et al. Soft robotic grippers. *Adv. Mater.* 30, 1707035 (2018).

Supplementary Table 16

Results from the System Usability Scale (SUS) questionnaires completed by four amputee subjects.

	Strongly Disagree	Disagree	Neutral	Agree	Strongly Agree
Frequent use		△	▲	□	■
Easy to use			△	▲	■□
Well-integrated functions				■	□▲△
Quick learning			△	■▲	□
Confident use			△	■□▲	
Unnecessarily complex	△	■▲	□		
Need technical support		■△	□	▲	
System inconsistency	□	■▲	△		
Cumbersome to use		■	□▲	△	
Significant learning effort	■	□▲△			

■Subject 1 □Subject 2 ▲Subject 3 △Subject 4

Supplementary Table 17

Results from the modified version of Quebec User Evaluation of Satisfaction with Assistive Technology (QUEST) questionnaires completed by four amputee subjects.

	Not satisfied at all	Not very satisfied	More or less satisfied	Quite satisfied	Very satisfied
Dimensions				■▲△	□
Weight				■□▲	△
Adjustments			□△	■▲	
Safety				△	■□▲
Durability		■	□▲	△	
Simplicity of use			□	▲△	■
Comfort	△	▲	■	□	
Effectiveness			▲△	□	■

■Subject 1 □Subject 2 ▲Subject 3 △Subject 4

Supplementary Table 18

NASA Task Load Index (NASA-TLX) test results for different execution modes of the bulb installation task.

	Low (0-30)	Low-med (31-50)	Medium (51-70)	High (71-85)	Very High (86-100)
Mental demand	-	-	①③	②	-
Physical demand	-	①	-	③②	-
Temporal demand	-	③	①②	-	-
Performance	-	②	③	①	-
Effort	-	-	①③	②	-
Frustration	-	①③	②	-	-

① Manipulation mode

② Grasp-only mode

③ Rigid neuroprosthesis
