# Supplementary material for: A high-dexterity soft neuroprosthetic hand for daily activities
Source: Nat Commun. 2026 Jul 4;17:8320. doi: 10.1038/s41467-026-75105-6 (PMC13470212; doi:10.1038/s41467-026-75105-6)
Supplement: Supplementary file 4 [file 41467_2026_75105_MOESM2_ESM.pdf]

Description of Additional Supplementary Files

File Name: Supplementary Video 1
Description: Mechanical design and dexterity demonstration.

File Name: Supplementary Video 2
Description: Demonstration of mechanical capabilities for dexterous manipulation.

File Name: Supplementary Video 3
Description: Demonstration of the sEMG acquisition and control algorithm performed by the amputee subject.

File Name: Supplementary Video 4
Description: Demonstration of continuous grasp and manipulation gestures by an amputee subject.

File Name: Supplementary Video 5
Description: Demonstration of a subject tuning the amplitude and strength of a specific grasp for objects with different dimensions and weights.

File Name: Supplementary Video 6
Description: Experimental process of the Box and Blocks Test.

File Name: Supplementary Video 7
Description: Experimental process of the Southampton Hand Assessment Procedure.

File Name: Supplementary Video 8
Description: Demonstration of versatile essential life skills.

File Name: Supplementary Video 9
Description: Demonstration of pinching slender rods with different diameters.

File Name: Supplementary Video 10
Description: Demonstration of continuous grasping of 20 different objects.

File Name: Supplementary Video 11
Description: Demonstration of bimanual coordination tasks.

File Name: Supplementary Video 12
Description: Demonstration of a female subject creating a hairstyle for her daughter.

File Name: Supplementary Video 13
Description: Demonstration of consecutive scissor cuts.

File Name: Supplementary Video 14
Description: Demonstration of two-object picking.

File Name: Supplementary Video 15
Description: Demonstration of bulb installation.

File Name: Supplementary Video 16
Description: Demonstration of amputee body movement during bulb installation tasks.
